# Nose-on-Chip Nanobiosensors for Early Detection of Lung Cancer Breath Biomarkers

**DOI:** 10.1021/acssensors.4c01524

**Published:** 2024-09-09

**Authors:** Vishal Chaudhary, Bakr Ahmed Taha, Lucky, Sarvesh Rustagi, Ajit Khosla, Pagona Papakonstantinou, Nikhil Bhalla

**Affiliations:** † Physics Department, Bhagini Nivedita College, University of Delhi, 110043 Delhi, India; ‡ Centre for Research Impact & Outcome, Chitkara University, Punjab 140401, India; § Department of Electrical, Electronic and Systems Engineering, Faculty of Engineering and Built Environment, Universiti Kebangsaan Malaysia, UKM, 43600 Bangi, Malaysia; ∥ Dr. B. R. Ambedkar Center for Biomedical Research, University of Delhi, 110007 Delhi, India; ⊥ School of Applied and Life Sciences, Uttaranchal University, Dehradun, Uttarakhand 248007, India; # School of Advanced Materials and Nanotechnology, Xidian University, Xi’an 710126, China; ¶ Nanotechnology and Integrated Bioengineering Centre (NIBEC), School of Engineering, Ulster University, 2-24 York Street, Belfast, Northern Ireland BT15 1AP, United Kingdom; ● Healthcare Technology Hub, Ulster University, 2-24 York Street, Belfast, Northern Ireland BT15 1AP, United Kingdom

**Keywords:** Biosensors, Nanotechnology, Lung cancer, Breathomics, Biomarkers, Nose-on-chip, Lab-on-chip, Early detection

## Abstract

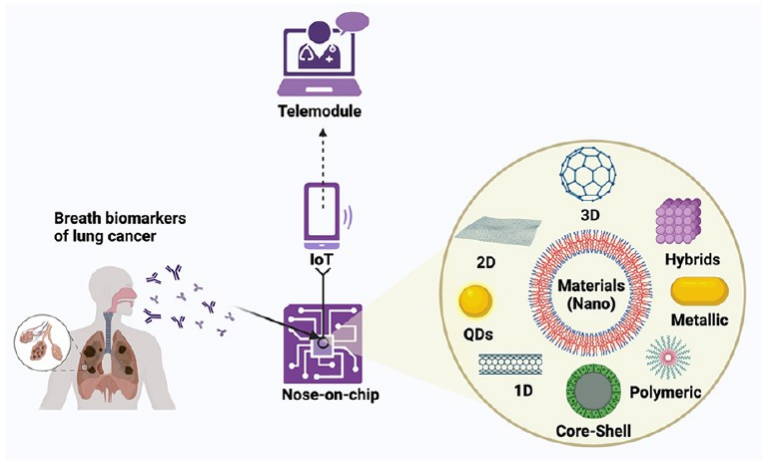

Lung cancer remains a global health concern, demanding the development of noninvasive, prompt, selective, and point-of-care diagnostic tools. Correspondingly, breath analysis using nanobiosensors has emerged as a promising noninvasive nose-on-chip technique for the early detection of lung cancer through monitoring diversified biomarkers such as volatile organic compounds/gases in exhaled breath. This comprehensive review summarizes the state-of-the-art breath-based lung cancer diagnosis employing chemiresistive-module nanobiosensors supported by theoretical findings. It unveils the fundamental mechanisms and biological basis of breath biomarker generation associated with lung cancer, technological advancements, and clinical implementation of nanobiosensor-based breath analysis. It explores the merits, challenges, and potential alternate solutions in implementing these nanobiosensors in clinical settings, including standardization, biocompatibility/toxicity analysis, green and sustainable technologies, life-cycle assessment, and scheming regulatory modalities. It highlights nanobiosensors’ role in facilitating precise, real-time, and on-site detection of lung cancer through breath analysis, leading to improved patient outcomes, enhanced clinical management, and remote personalized monitoring. Additionally, integrating these biosensors with artificial intelligence, machine learning, Internet-of-things, bioinformatics, and omics technologies is discussed, providing insights into the prospects of intelligent nose-on-chip lung cancer sniffing nanobiosensors. Overall, this review consolidates knowledge on breathomic biosensor-based lung cancer screening, shedding light on its significance and potential applications in advancing state-of-the-art medical diagnostics to reduce the burden on hospitals and save human lives.

Lung cancer (LC) is a primary global public health concern due to its pervasive manifestation and detrimental effects on humans, challenging present healthcare systems worldwide.^1−5^ It contributes to substantial morbidity and mortality and is the leading cause of global cancer-related deaths. According to the World Health Organization (WHO), there were approximately 2.21 million new cases of LC and 1.8 million mortalities attributed to it in 2020 alone.^4^ These figures accentuate the severity of the disease and its profound impact on a global scale, mandating the evaluation of its risk factors, timely diagnosis, and development of adequate treatment strategies.^2,3,6,7^

The primary risk factor for developing lung cancer is tobacco smoking, both active and passive.^8−11^ As per WHO, approximately 71% of total LC mortalities are attributable to smoking.^1^ Other potential factors that are a cause of LC are occupational hazards such as asbestos and radon exposure, air pollution, and genetic predisposition.^12−18^ The encumbrance of LC varies considerably across the globe and is influenced by socioeconomic factors, environmental conditions, and tobacco consumption patterns. For instance, developed countries historically reported higher incidence rates due to greater tobacco consumption rates. However, developing countries are now struggling with an escalating LC catastrophe as tobacco use and exposure to environmental risk factors increase.^19^ In these regions, limited access to healthcare, delayed diagnoses, and inadequate treatment facilities compound the challenges associated with managing lung cancer effectively. Moreover, the escalating issue of air pollution resulting from rapid urbanization and industrialization further contributes to the mortality associated with LC by compromising the respiratory, immune, and central nervous systems.

The substantial and devastating impact of lung cancer necessitates the implementation of preventive strategies and advancing early detection technologies. These strategies include reducing tobacco use, minimizing occupational exposures, mitigating air pollution, and raising awareness about the disease.^3^ Among these approaches, early diagnosis of lung cancer through screening programs and the development of innovative diagnostic techniques hold immense potential to enhance patient outcomes by facilitating timely interventions. Early detection of lung cancer through screening programs has demonstrated a significant positive impact on survival rates. For instance, low-dose computed tomography (LDCT) screening has shown promise in detecting lung cancer at earlier stages, which is more treatable. Early diagnosis and regular monitoring of lung cancer play a critical role in improving patient outcomes, underscoring the need for noninvasive and reliable diagnostic techniques.^2,3,20−23^

By prioritizing prevention strategies and investing in advanced diagnostic technologies, substantial strides can be made in combating the burden of LC, and the prognosis for individuals affected by this devastating disease can be improved. Several conventional methods are utilized to diagnose nose LC, encompassing imaging techniques, biopsy approaches, molecular testing, blood tests, and breath-based diagnostics.^11,24−28^ Imaging methods such as traditional computed tomography scans, magnetic resonance imaging, and chest X-rays effectively identify lung abnormalities and provide detailed visualization and characterization of lung tumors/nodules.

However, concerns arise regarding the potential risks of repeated radiation exposure to patients. Biopsy techniques, such as needle biopsy, bronchoscopy, and mediastinoscopy, are commonly employed to examine and identify cancerous cells. Still, these invasive procedures can be painful and distressing for patients, especially when repeated monitoring is required. Molecular testing, including genetic testing, next-generation sequencing, and blood tests that measure tumor markers like cytokeratin fragments and carcinoembryonic antigen, plays a crucial role in comprehensive tumor analysis.^29−32^ However, these methods can be resource-intensive and may not be readily accessible for repeated testing, highlighting the need for noninvasive alternatives (Figure 1). Furthermore, the impact of the COVID-19 pandemic has exacerbated challenges in lung care services, including diagnostics, due to overlapping symptoms with LC.^16,33,34^

**Figure 1:**
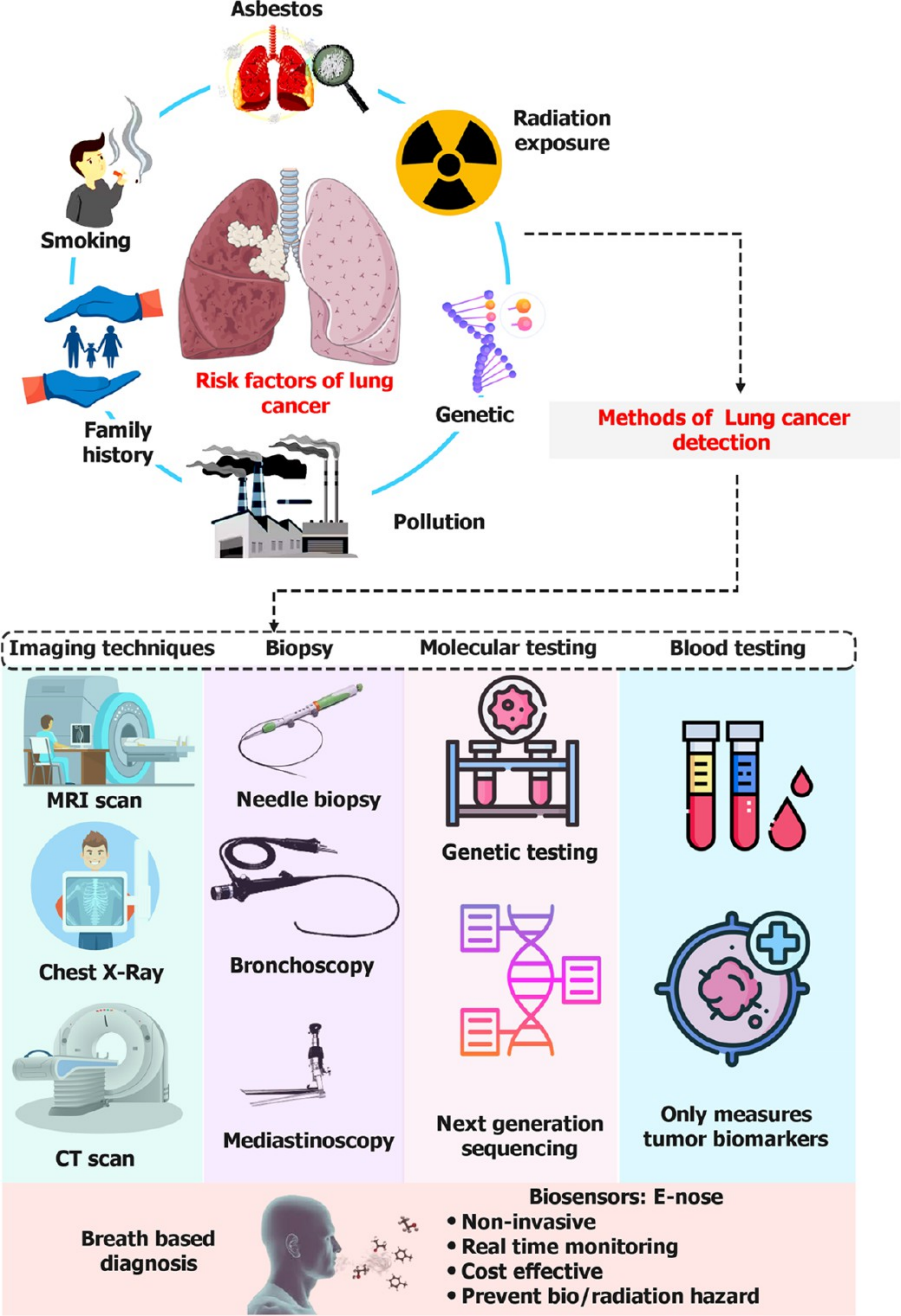
Overview of lung cancer diagnostics techniques from a conventional to modern age along with significant risk factors responsible for lung cancer.

Nonetheless, the global LC diagnostic market was calculated to be $879.6 million in 2021 and is anticipated to reach $1,853.04 million by 2031, with a compound annual growth rate of 7.7% from 2022 to 2031.^35^ The primary driving factor for this market growth is the ongoing research, development, and adoption of innovative noninvasive LC screening tools, which aim to reduce the likelihood of false negatives associated with existing tests, enable early tumor detection, and facilitate repeated monitoring with fewer resources and minimal stress on the patients.^36−38^ Among these noninvasive diagnostic strategies, breath-based diagnosis (BBD) has recently gained attention for its potential in early LC detection due to its simplified and patient-friendly procedure, noninvasive monitoring, and cost-effective and straightforward implementation.

## Emergence of Breath-Based Diagnosis as an Alternative Lung Cancer Screening Strategy

Breath analysis, known as breathomics, involves identifying and measuring various biomarkers such as gases, volatile organic compounds (VOCs), and biological components in exhaled breath.^26,39−47^ The VOCs serve as prominent biomarkers that reflect the metabolic and biochemical alterations associated with lung cancer and other prominent diseases such as asthma, pneumonia, irritable bowel syndrome, diabetes, liver disorders, neurodegenerative diseases, renal disorder, breast cancer, gastric disorders, *Helicobacter pylori* infection, and even coronavirus disease (COVID-19).^41,43,48−65^ BBD offers numerous advantages over conventional techniques, making LC screening more feasible. Unlike invasive procedures such as biopsies or blood tests, it is a non-invasive and painless procedure requiring only a breath sample.^11,37,59^ It allows for early detection with fewer human resources, enables rapid and real-time monitoring, and proves to be economically viable and patient-friendly.

Additionally, it helps prevent the bio/radiation hazards associated with other diagnostic methods while providing valuable insights into the prognosis of lung cancer.^24^ Breath analysis complements existing diagnostic techniques by providing additional information for accurate LC diagnosis and monitoring. It can potentially revolutionize LC diagnosis with its noninvasive nature, ease of use, and ability to provide valuable insights into the disease.^66−68^ The field of BBD for LC screening has witnessed significant advancements over time and can be marked by summarizing the following key milestones.^2,3,69,70^ In the early 1980s, scientists began exploring the use of breath analysis to detect VOCs as biomarkers for LC diagnosis. These investigations supported experimental outcomes using gas chromatography-mass spectrometry (GC-MS) for VOC scrutiny. In the 1990s, advancements in sensor technology and analytical techniques improved the sensitivity and specificity of breath analysis, yielding promising results in identifying specific VOCs associated with LC. The 2000s saw further research expansion, leading to the development of portable electronic nose (e-nose) devices that facilitated easier and faster VOC detection.^71−78^ Various sampling techniques were explored, including exhaled breath condensate (EBC) and offline breath collection methods.

**Figure 2:**
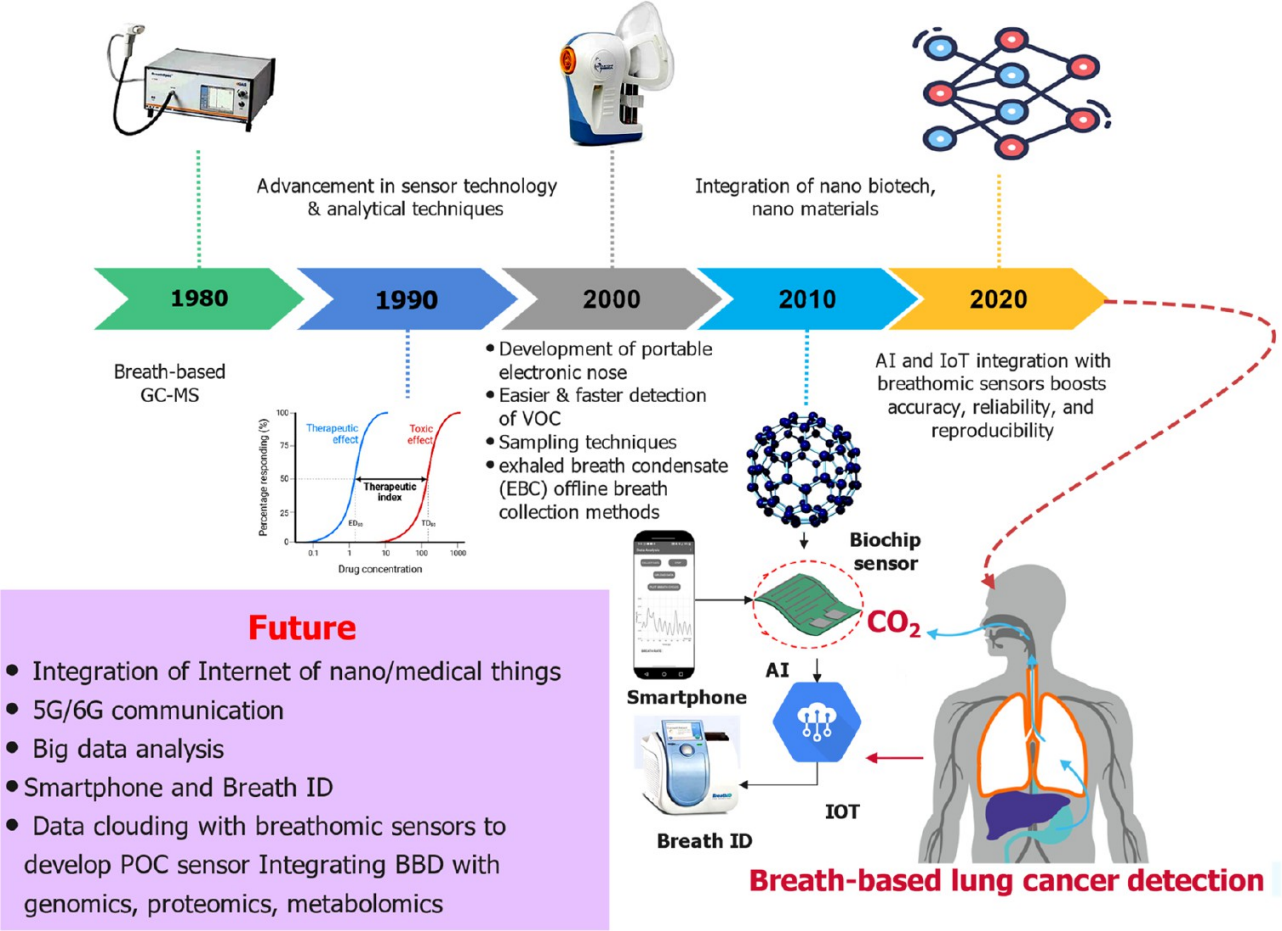
A decade timeline illustrates the development of breath-based detection of lung cancer and its prospects, along with critical milestones in lung cancer diagnostics development.

Further, integrating nanomaterials (NMs) in sensor technology revolutionized breathomic detection of LC, as nanoenabled VOC sensor arrays were developed, and the technology underwent validation through clinical trials in diverse populations.^47,79−92^ Moreover, integrating artificial intelligence (AI) with breathomic nanobiosensors has recently improved the results’ accuracy, reliability, and reproducibility.^93−103^ AI enables the standardization and validation of results through sampling protocols and diagnostic algorithms, expanding the clinical application of BBD. Ongoing research prospects involve integrating Internet-of-nanothings (IOT), Internet-of-medical-things, 5G/6G communication, big data analysis, and data clouding with breathomic sensors to develop point-of-care (POC) devices (Figure 2).^104−114^ These integrations of modern-age technologies with smart nanobiosensors have resulted in various POC modules, including Lab-on-chip (LOC) and Hospital-on-chip (HOC) nanobiosensors.^112,115−118^ These fifth-generation biosensors provide prospects of remote and personalized detection of various diseases, which are of ample importance in the era of the pandemic (COVID-19), where distant testing, minimal contact, and isolation are necessary for efficiently controlling the spread of infection.^95,112^ Enabling POC modules, such as LOC with breathomics, enables the detection of biomarkers of different diseases from human breath, which gives rise to Nose-on-chip (NOC) module-based nanobiosensors. These NOC nanobiosensors have the potential to screen diseases like LC, COVID-19, diabetes, renal disorders, irritable bowel syndrome, antimicrobial resistance (AMR), and neurodegenerative diseases through noninvasive and noncontact modes.^23,48−50,65,79,115,119−124^ It can potentially decrease the burden of present-day healthcare diagnostics and treatment facilities and provide medical aid, even in remote areas. These nanobiosensors have the potential to bring healthcare availability and equity irrespective of geographical, socioeconomic, or trained manpower constraints.^125^

Additionally, integrating BBD with other omics technologies, such as genomics, proteomics, and metabolomics, holds promise for a more comprehensive understanding of LC and improved diagnostic accuracy.^126−128^ Integrating these technologies, especially 6G networks and Organ-on-chip biosensors in smart POC modules, can revolutionize fifth-generation biosensor-enabled diagnostics and upgrade it to sixth-generation modules.^129−133^ For instance, adopting principles of quantum sensing and holography can lead to Surgery-on-chip modules, which can serve humanity better than previous-generation sensors and enhance patient outcomes.^115,125,132^ However, the detailed evaluation of fifth-generation nanobiosensors in BBD modules is still a matter of research for diseases such as LC and AMR.

Moreover, the field of BBD for LC screening is dynamic and continuously evolving as researchers strive to enhance its diagnostic capabilities and establish its clinical utility.^134−137^ This comprehensive review explores the current state of sensor-based breath analysis for LC diagnosis. It discusses the scientific basis of breath biomarkers, the various types of sensors employed, and their advantages, limitations, and potential for translation into routine clinical practice. By shedding light on the progress made and the challenges faced in this field, this review aims to contribute to the ongoing advancements in BBD and its potential to transform LC diagnosis.

## Biological Basis of Breath Biomarkers Related to Lung Cancer in Humans

This section elucidates the metabolic and biochemical alterations associated with LC that led to the production of specific biomarkers. It discusses the origin of these biomarkers and their relationship to tumor metabolism, inflammation, oxidative stress, and other pathological processes. The section emphasizes the potential of breath biomarkers as indicators of lung cancer presence, subtype, stage, and response to sensors.

### Breath Biomarkers in Human Breath for Lung Cancer Screening

In the realm of BBD of LC, extensive research has identified several biomarkers present in human breath that show potential as indicators of the disease. These biomarkers can be classified into distinct categories based on their chemical nature and origin, shedding light on the diverse molecular landscape associated with LC.^2,20,25,40,43^ Among all, VOCs/exhaled gases are most popular as breath biomarkers and represent a group of small organic molecules that readily evaporate and can be detected in exhaled breath. They arise from various sources, including the metabolic activities of tumor cells, oxidative stress, inflammation, and the breakdown of cellular components.^20,43,68,83,84^ Within the VOC category, specific compounds have been associated with LC, such as benzene derivatives (like toluene, benzene, and styrene), aldehydes (like acetaldehyde and formaldehyde), ketones (like methyl ethyl ketone and acetone), aromatic hydrocarbons (like xylene, ethylbenzene, and benzene), and nitrogen-containing compounds (like ammonia, nitrogen oxides).^23,45,79,85,89,138^ These VOCs provide valuable insights into the biochemical changes occurring in the lungs and hold potential as noninvasive biomarkers for early LC detection. These VOC concentrations are significantly higher in breath samples from LC patients than those from healthy individuals.

On the other hand, breath alkanes, another group of biomarkers, consist of saturated hydrocarbons found in exhaled breath. Research has shown that certain alkane compounds, including ethane and pentane, exhibit elevated levels in the breath of individuals with LC.^2,20^ These alkanes are believed to be linked to the peroxidation of unsaturated fatty acids in lung tissue, offering a glimpse into the lipid-related alterations associated with lung cancer development. The concentration of these breath alkanes is notably higher in LC patients than in healthy individuals.

Moreover, oxides of nitrogen, specifically nitric oxide (NO), have also been implicated in LC detection through breath analysis.^20,40,43^ Elevated levels of exhaled NO have been observed in individuals with LC, and this increase is thought to be a consequence of inflammation and oxidative stress within the lungs. Monitoring NO levels in breath samples may provide valuable information regarding the pathological processes occurring in lung cancer and aid in its early identification. The concentration of exhaled NO is significantly higher in LC patients than in healthy individuals. In addition, volatile sulfur compounds (VSCs) have emerged as potential breath-based biomarkers for LC screening.^20,40,43^ Methyl mercaptan (CH_3_SH) and hydrogen sulfide (H_2_S) are among the VSCs identified in the breath of LC patients. Altered sulfur metabolism in cancer cells is thought to contribute to the production of these compounds. The detection of VSCs in breath samples opens new avenues for exploring the metabolic changes associated with LC and developing noninvasive diagnostic approaches. The concentration of VSCs is also markedly elevated in breath samples from LC patients compared to that of healthy individuals.

By analyzing and understanding these biomarkers’ intricate profiles and concentrations, BBD for LC holds promise for improved early detection, allowing for timely interventions and enhanced patient outcomes. Continued research and technological advancements in this field are crucial for harnessing the full potential of breath analysis as a noninvasive and reliable tool in the fight against LC.

### Production of Breath Biomarkers of Lung Cancer in Humans

A range of intricate biological mechanisms influences the production of various biomarkers associated with LC in human breath. Metabolic alterations play a significant role in the generation of these biomarkers. In LC cells, metabolic changes occur due to dysregulation of cellular processes. Increased glucose metabolism, known as the Warburg effect, is a common feature of cancer cells, including LC.^20,40,43^ This heightened glucose metabolism can produce specific VOCs such as lactate, acetate, and pyruvate.^31^ These VOCs serve as impending biomarkers for early-stage detection and monitoring of LC.

Additionally, oxidative stress in LC cells can lead to lipid peroxidation, which generates volatile aldehydes and hydrocarbons.^20,40,43^ Aldehydes such as formaldehyde and acetaldehyde, as well as hydrocarbons like ethane and pentane, are among the volatile biomarkers associated with LC. The presence and concentration of these biomarkers in exhaled breath can provide valuable information about LC cells’ metabolic state and oxidative stress level. Inflammation and immune response also contribute to producing breath biomarkers in LC. Inflammatory mediators, including cytokines and chemokines, can stimulate the release of VOCs such as benzene derivatives (benzene, toluene, and styrene).^20,40,127^ These compounds can originate from various sources, including inflammatory cells and the breakdown of cellular components. Furthermore, reactive oxygen species (ROS) generated during inflammation and oxidative stress can promote the formation of VOCs, including aldehydes and ketones.^139^ These VOCs serve as potential indicators of the inflammatory processes associated with LC.

**Figure 3:**
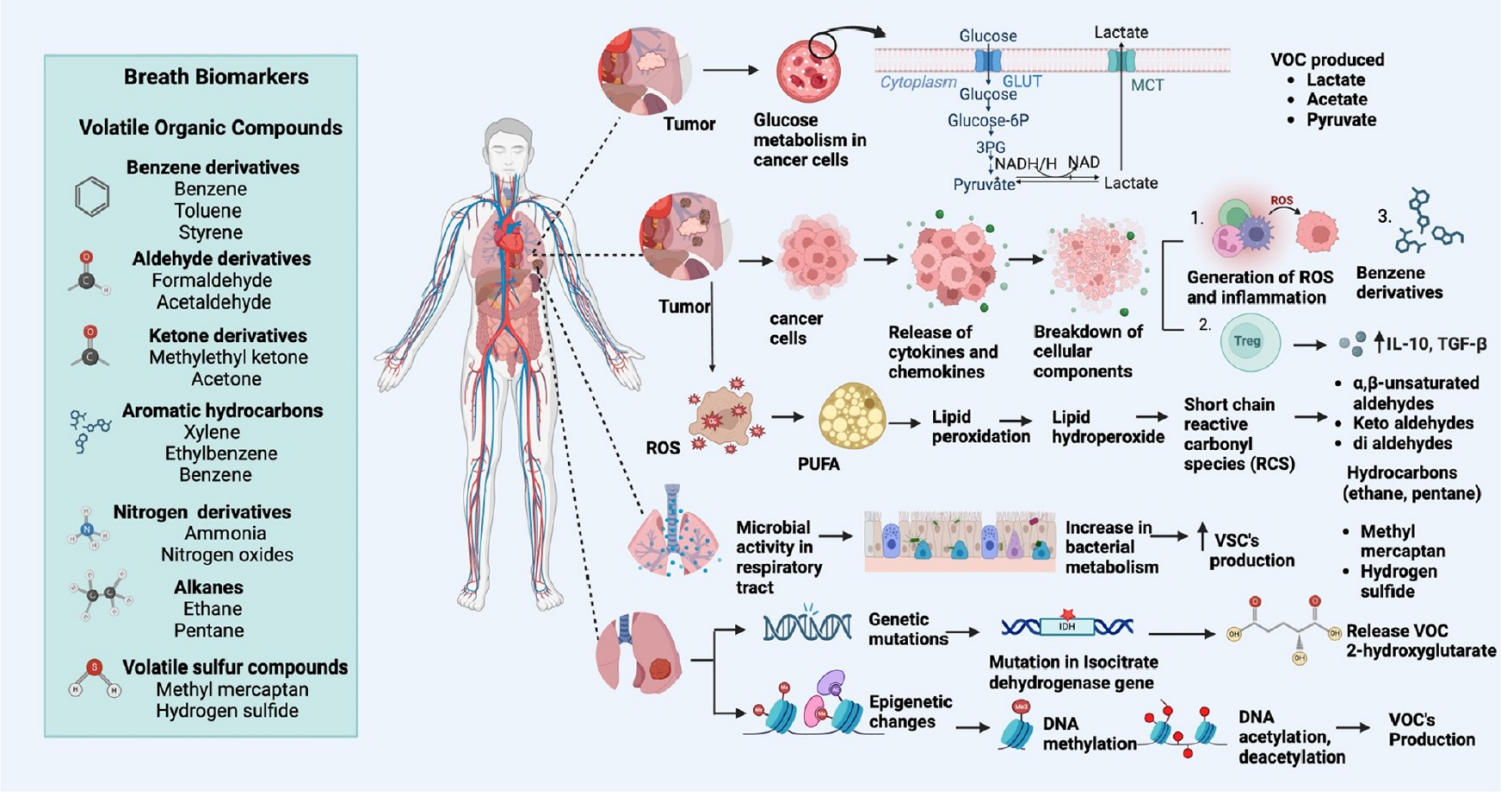
Key biomarkers found in human breath for detecting lung cancer, their structures, and their biological basis of evolution in the human body (created with BioRender.com).

Microbial activity within the respiratory tract is another factor influencing breath biomarkers in LC. Bacterial metabolism can contribute to the production of VOCs that can be detected in exhaled breath. Notably, VSCs have also been associated with LC.^140^ These VSCs are believed to arise from the metabolic activities of bacteria residing in the respiratory system. The presence of these VSCs in breath samples can provide insight into the microbial composition and activity within the lung microenvironment. Furthermore, specific genetic and epigenetic factors can impact the production of breath biomarkers in LC. Specific genetic mutations within LC cells can influence metabolic pathways and result in the production of distinctive VOCs.^141^ For example, isocitrate dehydrogenase (IDH) gene mutations can release VOCs like 2-hydroxyglutarate. Epigenetic changes, such as DNA methylation and histone modifications, are commonly observed in LC and can influence gene expression patterns, thereby affecting the production of specific VOCs. All biological bases of breath biomarker generation during LC due to different biological activities have been summarized in Figure 3.

Researchers can develop more targeted and sensitive diagnostic approaches by understanding the intricate biological mechanisms underlying the production of breath biomarkers in LC. These approaches can help in the early detection, monitoring, and personalized treatment of LC, ultimately improving patient outcomes and survival rates. However, it is important to note that the precise mechanisms and pathways by which VOCs are produced in the body, as well as their relationship with LC, are still being investigated. Identifying and validating specific VOCs as reliable biomarkers for LC diagnosis requires extensive research and large-scale studies. Understanding the metabolic, inflammatory, and genetic processes contributing to LC VOC generation can help in the development of targeted and sensitive detection methods. BBD holds promise for early detection, monitoring treatment response, and improving patient outcomes by analyzing the unique VOC profiles associated with LC.

## Emergence of Sensing Technologies to Detect Lung Cancer Biomarkers in Human Breath

This section provides an in-depth analysis of the different types of sensors employed for BBD of LC. It highlights traditional methods, including gas chromatography–mass spectrometry (GC-MS), and emerging sensor technologies, including metal oxide (MO) sensors, conducting polymers, surface acoustic wave sensors, and NM-based sensors. This section explores their working principles, sensitivity, selectivity, portability, cost-effectiveness, and suitability for clinical settings. To utilize the biomarkers for LC diagnosis, breath samples are collected and analyzed by using various techniques, including GC-MS, electronic nose (E-nose), and breathalyzer devices. These techniques offer different approaches to detecting and quantifying specific biomarkers in breath samples.^20,70,74,75^ For instance, GC-MS is a technique that separates and identifies volatile compounds in breath samples. It allows for the detection and quantification of specific biomarkers associated with LC. On the other hand, E-nose devices consist of sensor arrays that detect and differentiate volatile compounds based on their electronic responses. Pattern recognition algorithms are employed to analyze the sensor responses and identify the breath profiles associated with LC.

Breathalyzer devices, which are portable, utilize various sensor technologies such as metal oxide sensors or conducting polymers for LC screening and biomarker recognition.^56,127,142^ These devices detect and measure specific volatile compounds in breath samples. The collected data from breath analysis are compared with established reference ranges or specific breath profiles associated with LC to determine the likelihood of the disease’s presence or progression. Advanced statistical and machine learning (ML) algorithms can further enhance the accuracy and reliability of LC diagnosis using breath analysis by combining multiple biomarkers.^127,128^

**Figure 4:**
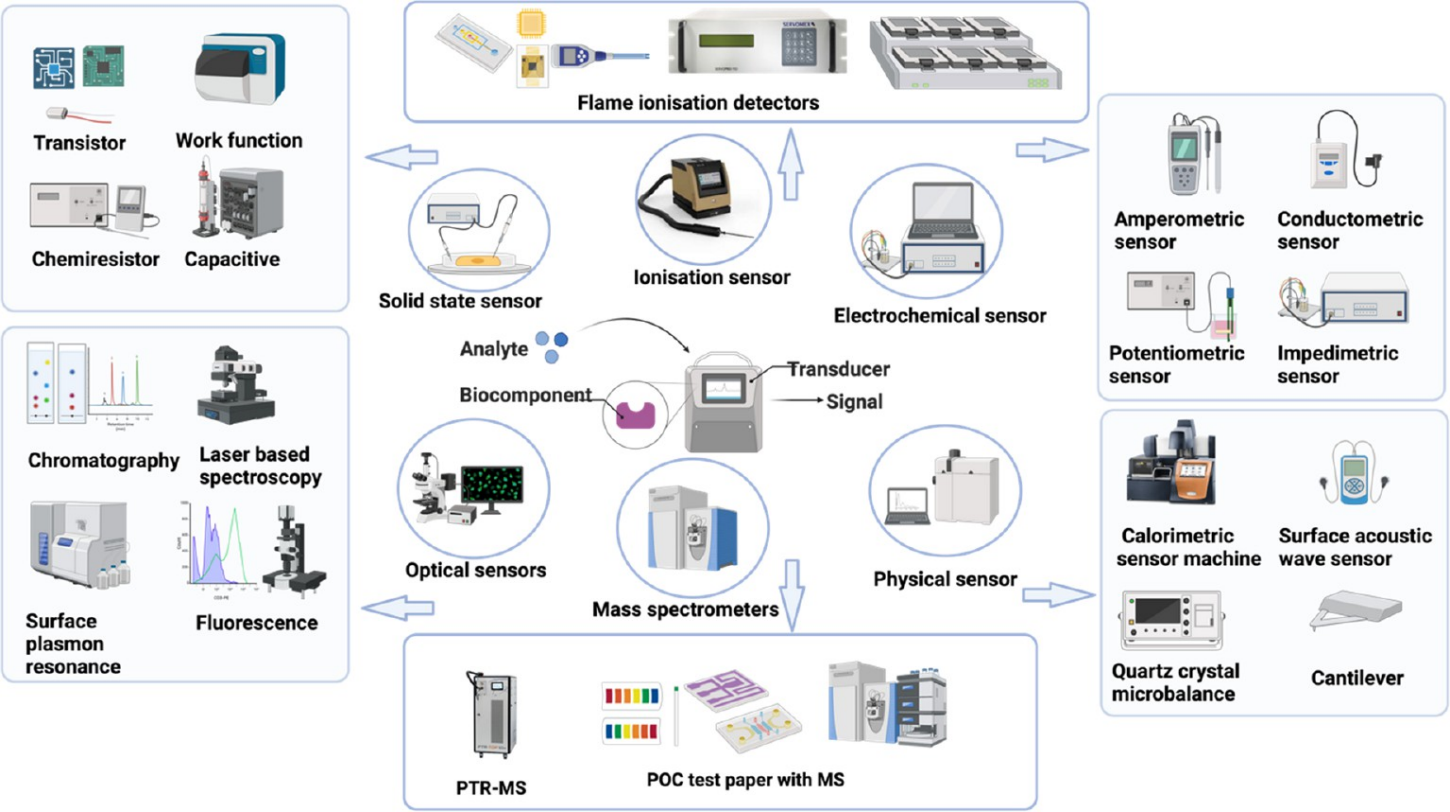
Various classes of biosensors and their fundamental detecting principles for the detection of various breath biomarkers for lung cancer screening. They include optical, mass-sensitive, physical, electrochemical, solid-state, and ionization-type biosensors with submodules based on the transducing principle (created with BioRender.com).

Different types of sensors are used in these strategies for LC diagnosis, each employing different principles to detect and analyze biomarkers in breath samples. These sensors generally consist of a sensing layer/material deposited over the substrate, which generates a sensing signal interacting with biomarker, transducer, microprocessor, and electronic components. They can be classified into solid-state sensors, gas chromatography (GC) sensors, optical sensors, electrochemical sensors, and mass spectrometers based on their transducing mechanism, and each class of sensor possesses diversified applications based on targeted merits and associated demerits (Figure 4).^2,24,25,27,56,142^

For instance, GC sensors, including flame ionization detectors (FIDs) and mass spectrometry (MS), are commonly utilized to measure the concentration of VOCs and are one of the pioneering technologies in BBD of human disorders. FID involves burning VOCs in a hydrogen flame and detecting the resulting ions, while MS identifies and quantifies VOCs based on their mass-to-charge ratios. For instance, Gregis et al.^143^ fabricated a microanalytical GC device to detect low concentrations of LC biomarkers in human breath with high selectivity even in the presence of common interfering analytes present in human breath, including carbon dioxide (CO_2_) and humidity. However, the complexity of the design, the requirement of preconcentration techniques, and the extended analysis time hinder their POC clinical applications for BBD of LC.

On the other hand, optical sensors utilize different mechanisms based on light–matter interactions, including surface-enhanced Raman spectroscopy, photoluminescence, fluorescence, surface plasmon resonance, and colorimetry. These sensors detect changes in the refractive index, emission of light, or color changes when VOCs interact with the sensor surface. For instance, Wang et al.^144^ validated the potential of the 2D platinum/titanium carbide MXene-carbon nanotube (Pt/Ti_3_C_2_T_*x*_-CNT) nanocomposite-based cataluminescence sensor for toluene detection, which is a prominent biomarker for BBD of LC within 1 s with rapid recovery in 30 s at 150 °C. The mechanism of toluene detection was based on its oxidation to form an excited-state CO_2_*, which produced a luminescent signal upon returning to its ground state. Besides, Nguyen et al.^145^ developed a novel multiarray biosensor with a gap plasmonic color film. The biosensor consisted of a layer of M13 bacteria placed between silver (Ag) nanocubes on a Ag film, enabling the detection of VOCs as LC biomarkers in exhaled breath. The breath samples from 50 LC patients and 70 healthy individuals were efficaciously categorized with an accuracy rate exceeding 89% using ML analysis. However, the need for a compact and portable design poses a challenge in developing a personalized device for LC screening, detection, and monitoring utilizing optical module sensors.

Electrochemical sensors, commonly utilized in breathalyzer devices, detect LC biomarkers by measuring the electrical currents generated by VOCs during specific electrochemical reactions. For example, Khatoon and colleagues^146^ presented an electrochemical sensor-based E-nose utilizing a cobalt/nickel (Co/Ni)-doped tin oxide (SnO_2_) nanosystem electrode to detect 1-propanol and isopropyl alcohol (IPA). The Co-doped SnO_2_ showed selectivity toward IPA, while Ni-doped SnO_2_ exhibited selectivity toward 1-propanol among other tested VOCs. Despite showing promise for early-stage LC screening through breath analysis, challenges such as the potential for false-positive or false-negative results, lengthy analysis time, and limited lifespan and stability impede their commercialization as POC devices for LC screening.

Mass spectrometers, such as PTR-MS, offer rapid real-time analysis and high sensitivity for the volatile organic compounds (VOCs)^147,148^. A recent study by Li et al.^148^ utilized a POC test paper with 4-ATP molecules as a probe to detect aldehydes in human breath, enhancing lung cancer screening. They employed thin-film reaction acceleration and coupled the test paper to a mass spectrometer by paper spray, achieving a high sensing response (0.1 parts per trillion: ppt) and a broad quantification range (10 ppt to 1 ppm: ppm). However, despite these advancements, there are limitations in mass-spectrometry-based breath analysis for lung cancer diagnosis, including sensitivity and specificity issues, standardization challenges, complex biomarker identification, heterogeneity of lung cancer, limited sample size, high costs, interference from unrelated compounds, and limited accessibility. Continued advancements are necessary to address these limitations and establish a breath-based diagnosis as a reliable tool for detecting lung cancer.

Among the diverse sensor types, solid-state sensors, including chemiresistive and quartz crystal microbalance (QCM) sensors, are widely used. QCM sensors detect changes in resonance frequency when VOCs adsorb onto a quartz crystal surface, offering highly sensitive and rapid detection.^149^ However, QCMs are yet to be explored in detail for BBD of LC. On the other hand, chemiresistor sensors detect changes in electrical resistance when VOCs interact with a sensor surface. They are cost-effective, simple, and suitable for portable devices, making them commonly used in E-nose devices for breath analysis, which will be discussed in detail in a subsequent section.

The selection of a specific sensor depends on factors such as sensitivity, selectivity, portability, cost, and requirements of the diagnostic application. Combining multiple sensor types or using sensor arrays can improve the detection accuracy and expand the range of detectable biomarkers. Chemiresistors, mainly, offer merits, such as cost-effectiveness, simplicity, and suitability for portable devices, making them a favorable choice over other techniques in specific applications. Overall, utilizing various techniques and sensor technologies, such as GC-MS, E-nose, breath analyzer devices, chemiresistors, and other solid-state sensors, enables detecting and analyzing biomarkers in breath samples for LC diagnosis. These approaches provide valuable insights into the presence and progression of LC, offering the potential for early detection and improved patient outcomes.

## Functional Nanomaterial-Based Biosensors Prospecting Nose-on-Chip Modules

Chemiresistors generally consist of a sensing layer deposited over the substrate with electrodes, an electronic transducer, and electronic circuitry such as Wheatstone bridge arrangement, packaging module, IOTs, and principal component analysis (PCA) analyzers for breath biomarker recognition. By engineering the sensory components, the efficacy of chemiresistors can be optimized and enhanced for selective detection of targeted analyte/biomarker.^110,150−152^ The primary component is its sensing layer, which has been engineered over the past few decades with different materials, morphologies, compositions, stoichiometry, physicochemical attributes, design, and architecture for enhancing the sensing characteristics of BBD.

The field of BBD for LC screening/monitoring has undergone a significant transformation with the advent of NMs, introducing exciting possibilities for developing susceptible and selective sensors. NMs possess several advantageous properties, such as a large effective surface area, customizable morphologies, diverse surface functionalities, and tunable physicochemical attributes. These characteristics enable NMs to serve as high-performance sensors, offering enhanced sensitivity, selectivity, recovery, and rapid response.^56,153−155^ One critical advantage of NMs is their superior effect surface area and distinctive morphologies, such as honeycomb structures or 2D stacks. These features increase the likelihood of interaction between biomarker molecules and the sensor surface, leading to a heightened and swift sensing response. Furthermore, the shallow penetration depth of biomarkers on the NM surface allows quicker recovery through easy desorption. Another remarkable aspect is the ability to tune the surface functionalities, stoichiometries, and physicochemical properties of NMs to target specific VOCs, thereby achieving superior selectivity and sensitivity. Consequently, NMs have emerged as a preferred choice for developing chemiresistive sensors to detect LC biomarkers in breath samples.

Various types of NMs have been extensively employed in fabricating breath sensors with NOC modules to diagnose LC. These include metal-based NMs, carbon-based NMs, organic NMs, advanced 2D materials, hybrids/nanocomposites, and biomaterials. Each class of NMs offers distinct advantages and characteristics that contribute to the overall effectiveness of breath chemiresistors in detecting LC biomarkers. However, most NMs have yet to be explored for BBD of LC and possess extreme potential to be tested as sensing platforms. This section highlights various classes of NMs used to design breath chemiresistors for the detection of LC biomarkers.

### Metal-Based Nanomaterials Enabled Sensors for Breath-Based Diagnosis of Lung Cancer

**Figure 5:**
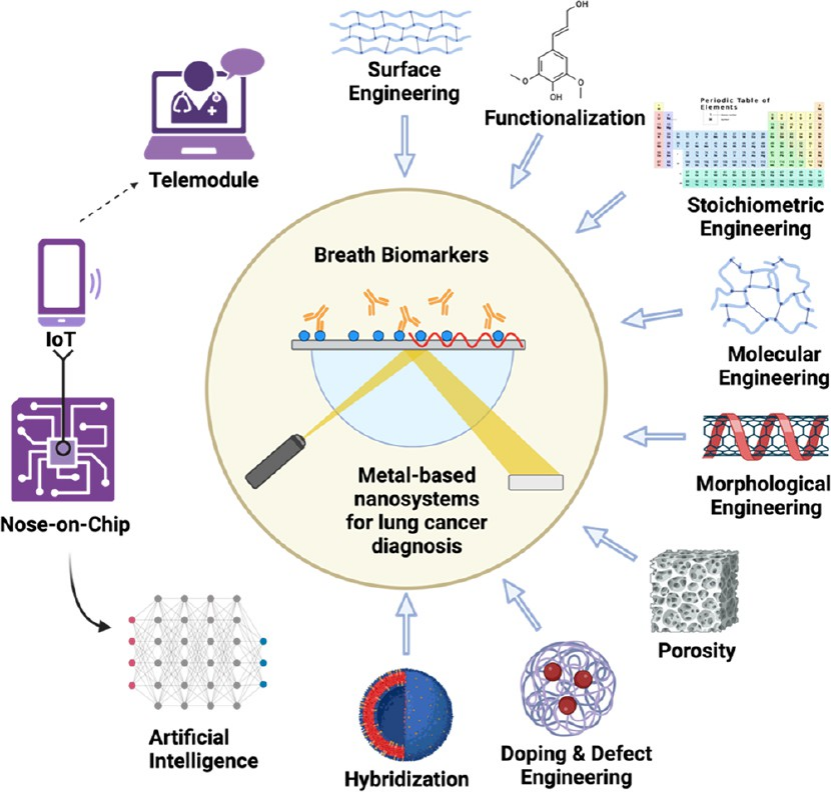
Schematic illustration of metal-based nanosystems with improved physicochemical attributes through different strategies, including functionalization, stoichiometric variations, molecular linkage, morphological variation, porosity inclusion, doping, defect engineering, and hybridization for enhanced detection of LC breath biomarkers in humans with smart and point-of-care modules (created with BioRender.com).

Metal-based (MB) NMs have emerged as a prominent class of nanosystems, enabling the development of highly effective nanosensors for BBD of LC. These nanosensors exhibit exceptional properties that make them well-suited for detecting LC biomarkers in breath samples.^56,57^ With their high surface-to-volume ratio, tunable physicochemical features, and diverse surface functionalities, MB NMs offer enhanced sensitivity, selectivity, and rapid response in capturing and detecting specific VOCs associated with LC. The unique properties of MB NMs, such as their optimizable electrical conduction behavior and catalytic activity, enable precise and reliable detection of biomarkers even at low concentrations. These attributes can be further enhanced and optimized with various strategies, including doping, additive manufacturing, tuning surface functionalities and band gap, optimizing morphology, and varying their stoichiometric configurations in the form of oxides, sulfides, and halides according to the targeted analyte/biomarker (Figure 5).

By strategically engineering MB nanostructures and tuning their topological functionalities, researchers have improved their sensing capabilities for accurate LC diagnosis. In their study, Peng et al.^156^ developed a chemiresistive sensor array consisting of gold nanoparticles (Au NPs) coated with different organic functionalities (dodecanethiol, 1-butanethiol, 2-ethylhexanethiol, hexanethiol, decanethiol, *tert*-dodecanethiol, 11-mercapto-1-undecanol, 4-methoxy-toluenethiol, and 2-mercapto benzoxazole). The aim was to detect and analyze VOCs in exhaled breath, enabling the diagnosis of LC based on breath analysis. Initially, using GC-MS and solid-phase microextraction, they identified 33 general VOCs and nine infrequent VOCs, which were present in at least 83% of LC patients but in fewer than 83% of healthy individuals. Through optimization and training using PCA, four VOCs were identified as highly consistent among simulated and patient breath samples, demonstrating excellent sensitivity, selectivity, repeatability, and stability. The incorporation of organic functionalization on Au NPs (size: 5–10 nm) played a crucial role in enhancing VOC selectivity, robustness, and sensor fabrication. This research presented potential for noninvasive and early detection of LC, potentially improving patient outcomes and enhancing clinical management. These findings provide a solid foundation for further advancements in the field.

Following this, Zhao and colleagues^157^ conducted a study where they developed a chemiresistor array consisting of molecularly linked Au NPs. The array demonstrated the ability to detect a mixture of VOCs and LC breath biomarkers, achieving a low detection limit (LOD) of 20 ppb for acetone detection. Importantly, this capability showed promise in distinguishing breath samples from LC patients and healthy individuals, even under normal operating conditions. These results provide a strong foundation for further investigations, including testing more breath samples from both patient groups. This additional research will contribute valuable insights toward developing a point-of-care device for breath monitoring in LC patients, offering improved diagnostics in the future.

**Figure 6:**
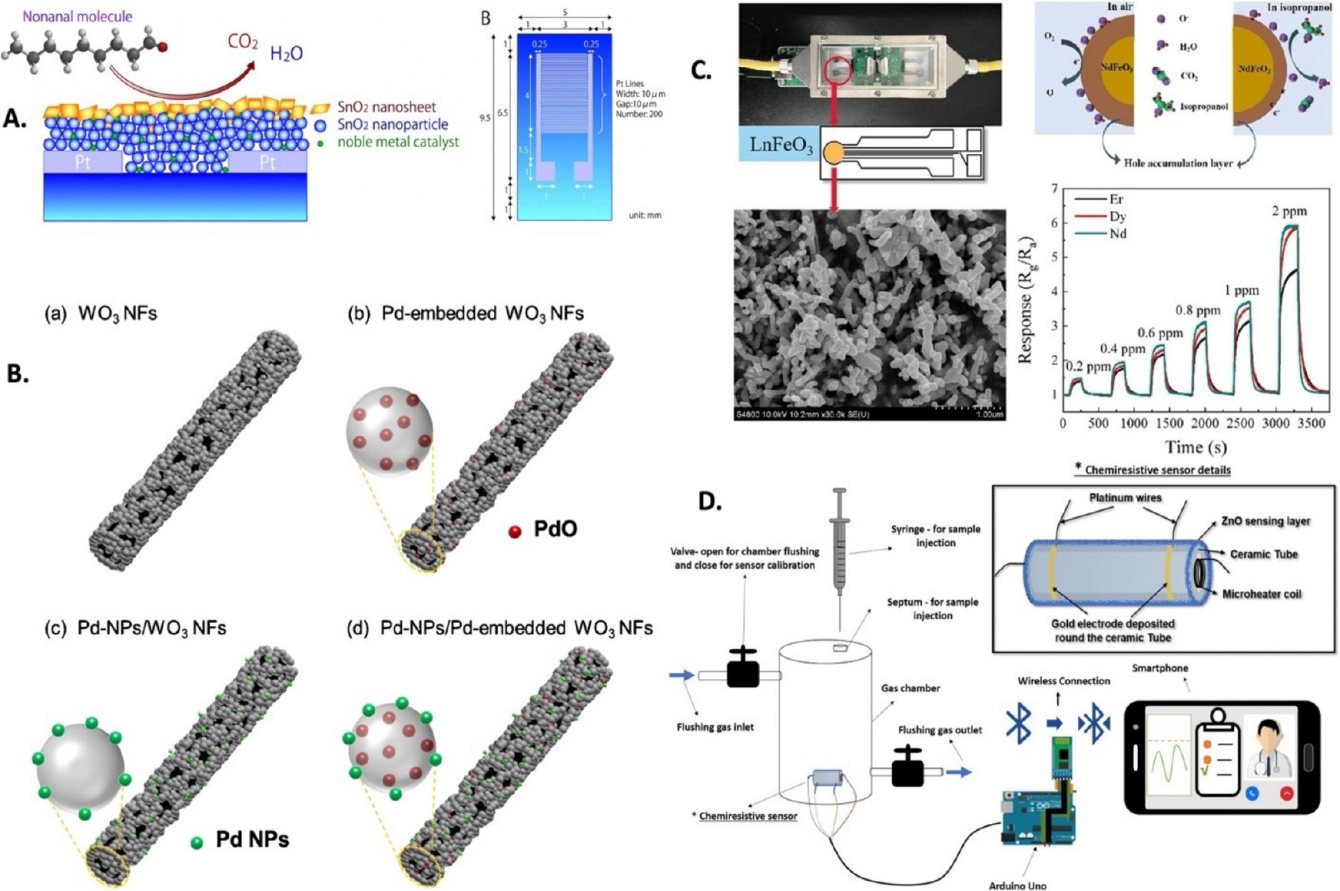
Detection of prominent breath biomarkers of lung cancer using metal-based nanosystems, including (A) variation in morphologies of SnO_2_ nanosystems with functionalization with the metal catalyst for detection of nonanal molecules and a designed chemiresistor based on these nanosystems. Reproduced from ref (158). Copyright [2022] Elsevier. (B) Surface modification and doping strategies to enhance the biomarker detection capability of WO_3_ nanofibers, including embedded Pd, Pd nanoparticles, and both. Reproduced from ref (163). Copyright [2014] Elsevier. (C) Chemiresistor fabricated using LnFeO_3_ with distinct morphology, detection mechanism, and sensing characteristics for detection of isopropanol. Reproduced from ref (163). Copyright [2022] Elsevier. (D) Chemiresistive smart sensor based on the ZnO nanosystem for detection of lung cancer biomarkers with the integration of Arduino Uno, Bluetooth, and mobile phone resulting in a point-of-care and personalized module. Reproduced from ref (169). Copyright [2021] Elsevier.

Further, the variation of stoichiometric composition with MB NMs as oxides, sulfides, and halides were performed and are among the most commercial class of sensors due to high ambient stability and tunable physicochemical attributes. Metal oxide-based (MO) nanosystems with distinct morphologies have exhibited promising outcomes in detecting LC through breath analysis. In a study, Masuda et al.^158^ fabricated a chemiresistor enabled by SnO_2_ nanosheets to detect nonanal gaseous biomarkers in human breath for LC screening (Figure 6A). The interaction of nonanal molecules via adsorption on SnO_2_ nanosheets resulted in a change in sensor resistance due to a surge in the carbon number of aldehyde, which resulted in molecular level detection of aldehyde by fabricated nanosystems and effective LC screening. Moreover, combining two different morphologies with metal catalysts in single nanosystems improves their sensing performance. In another study, Masuda et al.^159^ developed a sensor to identify 1-nonanal gas, a specific biomarker in the breath of individuals with LC. The sensor design combined SnO_2_ nanosheets, SnO_2_ NPs, and noble metal catalysts. The resulting nanosensor displayed a significantly enhanced sensitivity of 1.12 within a concentration range of 1 to 10 ppm and high recovery rates at 300 °C. The improved sensitivity can be ascribed to the augmented oxidation of 1-nonanal molecules facilitated by SnO_2_ nanosheets with the (101) crystal faces. This innovative approach offers a straightforward and efficient method for early detection of LC, whereas it is limited in the context of high-temperature operation.

The doping of MO with metals regulates their physicochemical features according to the targeted biomarker. Guntner et al.^160^ evaluated the LC screening potential of E-nose based on Pd-, Ti-, Pt-, and Si-doped SnO_2_ nanostructures by detecting low traces of formaldehyde. The fabricated E-nose exhibited stable sensing responses, a low LOD as low as three ppb, and a signal-to-noise ratio (SNR) less than 25 at breath-realistic 90% relative humidity (RH), making it eligible for LC screening. The doping of metals enhances the sensing signal by increasing the catalytic activity. Similarly, Luo et al.^161^ studied the Fe-doped zinc oxide (ZnO) nanoneedle-based chemiresistor to detect trace-level isopropanol (below ten ppm). The study revealed that the doping of Fe considerably augmented the sensing behavior of ZnO nanoneedles toward isopropanol. The Fe doping concentration was optimized to 5 wt % for isopropanol sensing, and the optimum operational temperature is 275 °C. The chemiresistor exhibited a higher sensitivity toward 250 ppb isopropanol, composed with higher stability under variable relative humidity and suitability for LC diagnosis through breath isopropanol detection.

Moreover, the metal-functionalized MO nanosystems have also shown potential for BBD of LC due to their catalytic and synergistic merit. For instance, Shim et al.^162^ designed a chemo-resistive sensor for sensitively monitoring trace concentrations of ethanol and NO_2_ using cross-linked nanodomes of WO_3_ decorated with Au. These nanodomes exhibited exceptionally high sensitivities, selectivity, and LOD in the PPT range for NO_2_ at 250 °C and ethanol at 450 °C. The enhanced sensing performance of these nanodomes can be attributed to the presence of Au decoration, which depends on the specific target gas and involves a complex interplay between electronic and chemical sensitizations. These findings demonstrate the potential of Au-decorated WO_3_ nanodomes for screening LC and asthma, highlighting their suitability for accurate and reliable detection in these medical applications.

Likewise, Kim et al.^163^ designed a highly selective and sensitive H_2_S/toluene sensor enabled by Pd-functionalized WO_3_ nanofibers for BBD of LC (Figure 6B). The Pd-loaded nanofiber-based sensor demonstrated a high toluene sensitivity (of around 5.5 at 1 ppm) and notable selectivity toward H_2_S (sensitivity = 1.36 at 1 ppm) at 350 °C compared to pure WO_3_, which was ascribed to the catalytic action of Pd over the WO_3_ surface. The breath toluene concentration in LC patients is around 80–100 ppb, which is two/three times enhanced compared to the concentration in healthy people’s exam; ed breath (20–30 ppb). These remarkable sensing features with an LOD of 20 ppb exhibit the potential of a functionalized nano-MO system as a promising candidate for breath-based LC monitoring.

Similarly, Zhang et al.^164^ studied the possibility of LC screening by detecting low-trace breath isopropanol using Ag NP decorated indium oxide (In_2_O_3_) hollow sphere-based sensors. The high performance of the fabricated sensor was ascribed to the formation of the Schottky barrier at the interface of Ag/In_2_O_3_ and the catalytic action of Ag NPs. In recent studies, researchers have explored the use of advanced hybrid metal oxide NPs with high porosity to develop sensing platforms for detecting LC biomarkers in human breath. For example, Yang et al.^165^ presented an innovative chemiresistor based on hierarchical porous lanthanum ferrite (LaFeO_3_) NPs. The sensor exhibited a high sensitivity of 116 toward formaldehyde at a level of 50 ppm and operated at 125 °C. Additionally, it demonstrated a low LOD of approximately 50 ppb. The improved sensitivity of the nanosystem can be ascribed to its hierarchical porous structure, which provides a high surface area and pore volume for efficient analyte capture. These findings highlight the potential of hierarchically porous NPs as an auspicious material for the growth of breathomic sensors for LC diagnosis.

In a separate investigation, Chai et al.^166^ explored the potential of three types of LnFeO_3_ (where Ln represents Nd, Dy, and Er) for the early detection of LC through sensing isopropanol in exhaled breath (Figure 6C). The developed sensor demonstrated excellent sensitivity, stability, and selectivity at 275 °C, even under high humidity conditions, with an LOD of 0.2 ppm. The outstanding gas-sensing performance of LnFeO_3_ can be attributed to its large surface area and the low bond energy between Ln series elements and oxygen ions, allowing for easy breaking and reaction.

In a recent study conducted by Li et al.,^167^ an innovative sensor utilizing delafossite silver chromate (AgCrO_2_) NPs was demonstrated for the detection of ultralow levels of *n*-propanol in human breath, with an LOD as low as 100 ppb, for LC screening. The performance of AgCrO_2_ was compared to that of copper chromate (CuCrO_2_) and commercial SnO_2_, revealing superior characteristics, including higher selectivity, dynamic response, and logarithmic linearity, while operating at a lower working temperature. The sensing mechanism was elucidated through a combination of first-principles calculations and energy band theoretical investigation, indicating that the exceptional sensitivity of AgCrO_2_ to *n*-propanol stems from the chemical adsorption of gaseous molecules onto the Ag surface after dehydrogenation on the Cr surface of AgCrO_2_. These findings emphasize the significance of utilizing MO NMs with low binding energy and dehydrogenated MOs in detecting LC using human breath analysis.

In addition, researchers have explored the use of zeolite-functionalized MO NPs to improve the detection of LC biomarkers in human breath. An example is the work by Kumar et al.,^168^ who integrated dealuminated zeolite with SnO_2_ NPs to detect LC biomarkers, including propanol, formaldehyde, and toluene, at low concentrations as low as 200 ppb. The chemiresistor developed in this study demonstrated remarkable performance in monitoring propanol, exhibiting a sensitivity of approximately 96 ± 2% within a rapid response time of approximately 10 ± 1 s at an operating temperature of 275 °C. The zeolite functionalization unveiled excellent catalytic activity in the dehydration of propanol molecules, resulting in propene production. This approach holds promise for enhancing the detection efficiency of LC biomarkers through the synergistic effects of zeolite and metal oxide nanoparticles.

Recently, integrating MB nanosystems with advanced technologies such as miniature processors, AI, and IOTs has enabled on-site/lab-on-chip disease screening using breathomic sensors.^112^ Recently, Salimi et al.^169^ conducted a study utilizing ZnO nanosheets to develop a smartphone-integrated POC biosensor/chemiresistor for noninvasive BB of LC (Figure 6D).

Their research focused on detecting two primary LC biomarkers, acetone and isopropanol, with remarkable LODs of 4 and 11 ppb, respectively. The nanosheets exhibited rapid detection, prompt recovery, and excellent selectivity, even in the presence of interfering analytes commonly found in human breath, such as humidity and CO_2_. The device was operated using a POC module that integrated an Arduino Uno with Bluetooth capability, enabling the sensor’s signal to be read and data transmitted to a smartphone. In healthy subjects, the acetone concentration ranged from 41.6 to 1709.8 ppb, while for individuals with LC, it varied from 112.3 to 2653.7 ppb. Similarly, the concentration of 2-propanol varied from 0 to 506 ppb in healthy subjects and from 8.7 to 989 ppb in individuals with LC. These findings highlight the promising potential of the developed POC sensor for on-site diagnosis of LC, pending further validation through clinical trials and data analysis.

### Advanced 2D Materials as a Sensing Platform for Breath-Based LC Screening

Apart from MB NPs/MO NPs, other inorganic materials, including advanced two-dimensional (2D) materials, have shown potential for LC biomarker detection in human breath. For instance, molybdenum disulfide (MoS_2_) based chemiresistors have emerged as a promising technology for the BBD of human disorders. These chemiresistors utilize the unique properties of MoS_2_, a 2D material known for its high surface area and sensitivity to target analytes. By detecting changes in the electrical resistance of the MoS_2_ chemiresistor upon exposure to VOCs present in exhaled breath, biomarkers associated with LC can be identified. For example, in a study conducted by Kim et al.,^170^ a chemiresistor based on MoS_2_ was modified with a thiolate ligand called mercaptoundecanoic acid (MUA) to detect LC breath biomarkers. The MoS_2_ chemiresistor exhibited positive responses to oxygen-functionalized VOCs, while the MUA-conjugated MoS_2_ chemiresistor displayed negative responses to the same VOCs. This successful ligand conjugation demonstrated the enhanced functionality of the MoS_2_ matrix to detect LC. The fabricated MoS_2_ sensors demonstrated high sensitivity, detecting representative VOCs at concentrations as low as 1 ppm. This tunable and sensitive VOC sensor holds excellent promise for real-world applications in LC diagnosis through breath analysis.

**Figure 7:**
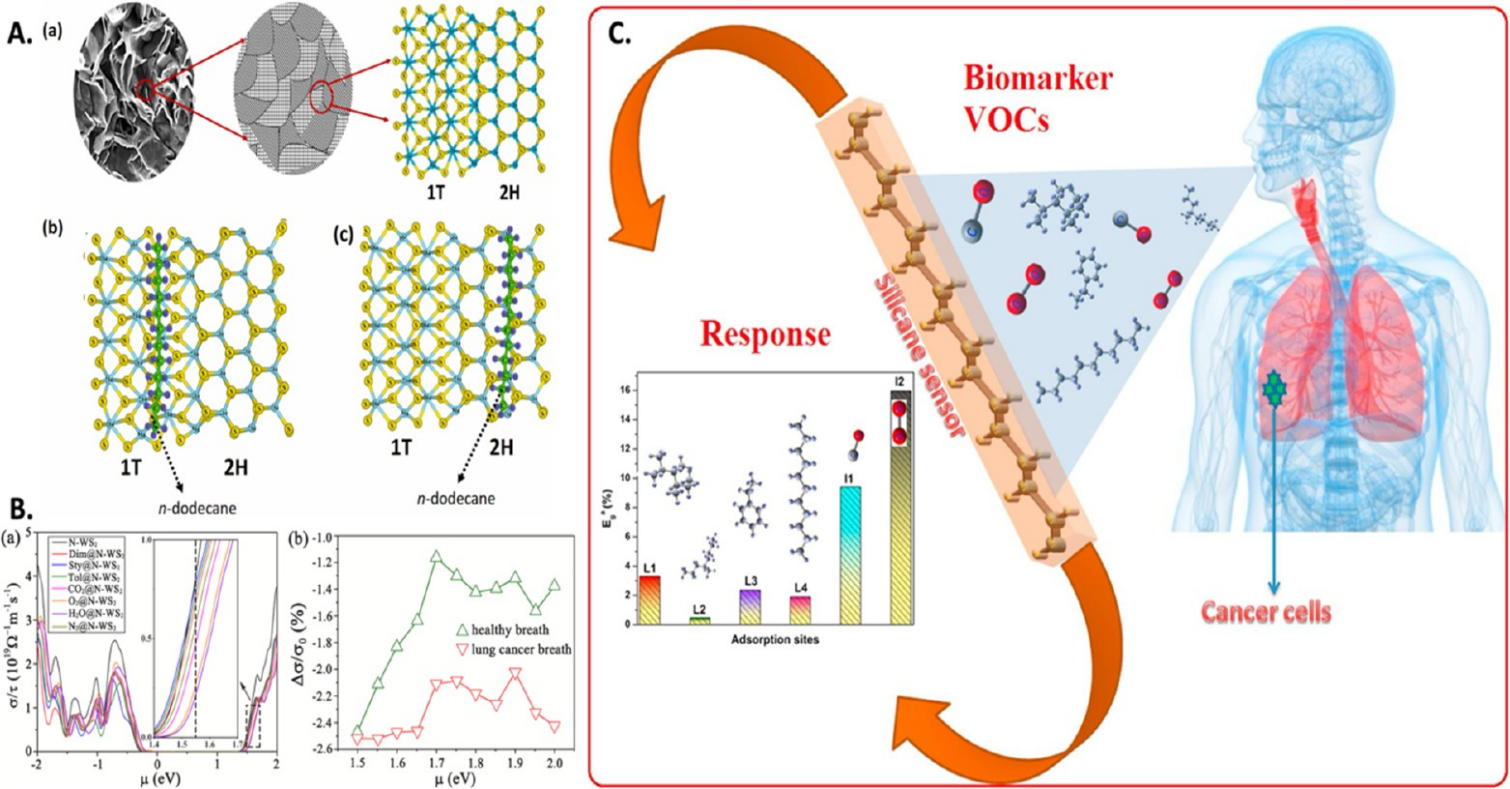
Potential of advanced 2D inorganic materials for the detection of breath biomarkers of LC including (A) multiphase MoS_2_ and illustration of the sensing mechanism of *n*-dodecane by it from different views using DFT analysis. Reproduced from ref (173). Copyright [2023] Elsevier. (B) Variation in electrical conductivity of nitrogen-doped WS_2_ in response to the breath of LC and healthy individuals, illustrating its potential to detect breath biomarkers of LC using a chemiresistive module. Reproduced from ref (174). Copyright [2023] Elsevier. (C) Illustration of the utilization of the 2D-silicone-based chemiresistor to detect various breath biomarkers of LC and their selectivity and sensitivity studies. Reproduced from (175). Copyright [2018] Elsevier.

Zhao et al.^171^ further calculated the Ni doping effect on MoS_2_ sensor performance for detecting breath biomarkers in early-stage LC screening. The density functional theory (DFT) estimations revealed that the Ni doping resulted in dramatic modification in geometric and electronic attributes of the MoS_2_ sensor, which resulted in its superior performance compared to the pristine MoS_2_-based sensor. Recently, Chhetri et al.^172^ revealed through DFT calculations the enhanced adsorption of two LC biomarkers, including isobutyraldehyde and methylcyclopentane, over the MoS_2_ monolayer, which supports the experimental findings of previous studies and makes MoS_2_ an eligible candidate for LC screening through breath analysis. Moreover, Muthumalai et al.^173^ experimentally verified the potential of a multiphase MoS_2_-based chemiresistor to detect nonpolar *n*-dodecane, a prominent LC biomarker in human breath (Figure 7A). The sensor exhibited high sensitivity toward low trace *n*-dodecane as low as 400 ppb with rapid response/recovery within 40/60 s, respectively. Supporting it, DFT outcomes show that the manifestation of the metallic 1 T phase in the multiphase MoS_2_ is accountable for biomarker/VOC adsorption and charge transferal during the *n*-dodecane monitoring. These studies revealed the potential of MoS_2_-based chemiresistors for early-stage LC screening.

Recently, 2D transition metal dichalcogenides (TMDs) have been explored as sensing platforms for designing chemoreceptors for breath analysis. Recently, Li et al.^174^ theoretically explored the possibility of a nitrogen-doped tungsten disulfide (N-doped WS_2_) based nanosensor for BBD of various LC breath biomarkers, including 2,3-dimethylhexane, styrene, and toluene (Figure 7B). DFT calculations revealed assertive adsorptive, recoverable, humidity-resistant, and selective sensing behavior of these biomarkers at room temperature on N-doped WS_2_. It was attributed to N-doping, which considerably improved the adsorption strengths of low-trace LC breath biomarkers, making it eligible to design early-stage LC screening.

Apart from MB/MO/2D-MB nanosystems, 2D metalloids and insulators have also been observed for BBD in human disorders. Nagarajan et al.^175^ has presented a theoretical prediction regarding the potential use of hydrogen-deficient 2D silicane nanosheets in detecting various biomarkers associated with LC, such as 4-methyl octane, ethylbenzene, 2,3,4-trimethyl hexane, and undecane (Figure 7C). The sensitivity of these biomarkers to hydrogenated silicane has been verified using the DOS spectrum. The investigation reveals that hydrogenated silicane nanosheets demonstrate optimal sensitivity to these specific biomarkers found in the exhaled breath of LC patients. These findings strongly suggest that hydrogenated silicane nanosheets have significant potential for application in the detection of LC disease.

In a recent study by Mashhadbani et al.,^176^ the authors explored the potential of single-layer topological insulator armchair stanene nanoribbons for LC screening through theoretical analysis using DFT and nonequilibrium green function estimations. The research findings revealed that armchair stanene nanoribbons exhibited significantly higher adsorption energy than various reported materials, including CNTs, phosphorene, and silicene. The outcomes also indicated that configurations featuring a single vacancy and edge defects in the nanoribbons improved the sensitivity and selectivity of the sensor due to the presence of free dangling bonds. Specifically, armchair stanene nanoribbons with edge defects on both sides were found to reduce the adsorption energy to −8.35 eV and achieve a sensitivity increase of up to 45% for detecting toluene. These results suggest that these 2D materials based on metals, metalloids, or insulators are promising candidates for LC screening. However, it is essential to highlight that the experimental evaluation of these novel NMs for LC screening needs to be improved, and further investigations are required.

### Carbon-Based Nanomaterials as Sensing Platforms for Breath-Based LC Screening

**Figure 8:**
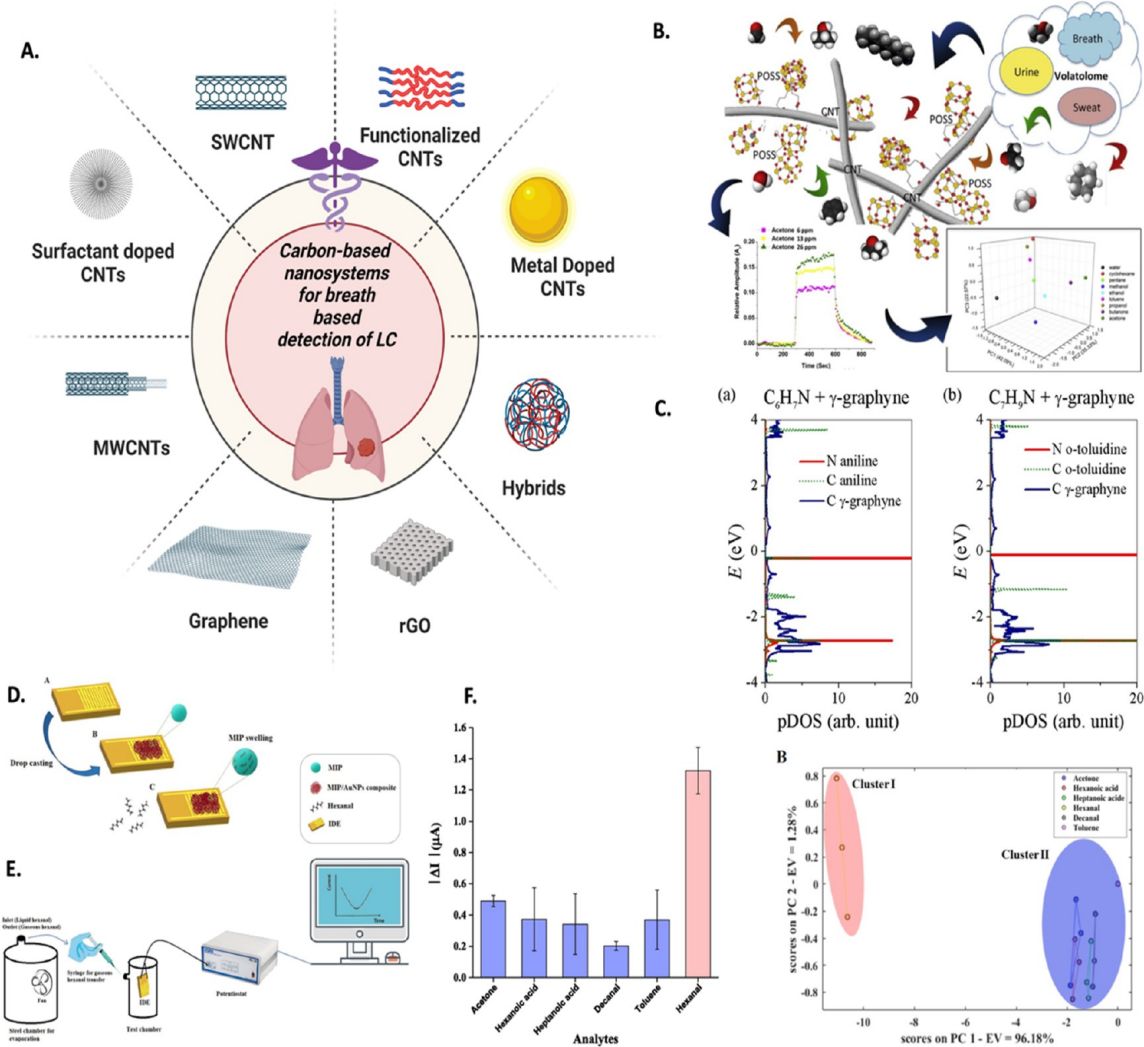
(A) Carbon-based nanosystems including functionalized, doped, metal-decorated, and hybridized SWCNT, MWCNT, graphene, and rGO for detection of breath biomarkers for LC (created with BioRender.com). (B) Quaternum chemiresistor based on various polyhedral oligomeric silsesquioxane functionalized CNTs for detection of LC biomarkers in human breath with sensing characteristics. Reproduced from ref (179). Copyright [2016] Elsevier. (C) The density of states variation in aniline and *o*-toluidine adsorbed γ-graphyne with the potential to detect LC biomarkers from human breath. Reproduced from ref (183). Copyright [2020] Elsevier. MIP-based chemiresistors for LC diagnosis: schematic illustration of (D) a Au nanoparticle functionalized MIP-based sensory chip fabricated through drop casting strategy, (E) hexanal sensing setup to analyze sensing performance of fabricated MIP, and (F) sensing characteristics of a Au doped MIP-based chemiresistor toward breath biomarkers of LC. Subfigures D, E, and F are reproduced with from ref (186). Copyright [2022] Elsevier.

2D/1D carbon-based NMs, including reduced graphene oxide (rGO), graphene oxide (GO), graphene, and CNTs, have also been experimentally evaluated for LC biomarker detection capabilities (Figure 8A). For example, Peng et al.^177^ fabricated an array of chemiresistors using nonpolymeric organic material coated single wall CNTs (SWCNTs) for potential LC screening through breath evaluation. PCA analysis showed distinguished signals between healthy and cancerous breath signals, which showed potential for early detection using nonpolymeric organic material coated single wall CNTs (SWCNTs) for potential LC screening through breath evaluation. PCA analysis showed distinguished signals between healthy and cancerous breath signals, which showed potential for early-stage diagnosis of LC. Further, Chatterjee and colleagues^178^ observed the tailoring of desired selectivity and sensitivity of the sensor toward LC breath biomarkers by varying the nature of the surfactant during the fabrication of CNTs. It was revealed that the sensing performance of surfactant-assisted CNTs depends upon the interaction of surfactant present as a dopant in CNTs, their supramolecular arrangement with CNTs, the electrical conductivity of CNT–surfactant nanosystems based on optimizing the precursor’s concentration, and optimization of its physicochemical attributes. The outcomes revealed that sodium deoxycholate-assisted CNT is responsive to alcohols and water and the triton X-405-mediated CNT toward benzene, *n*-pentane, and chloroform; sodium dodecylbenzenesulfonate-mediated CNT was not responsive toward biomarkers, benzalkonium chloride-assisted CNT toward *n*-pentane, isoprene, acetone, and ethanol; and 1-hexadecyl trimethylammonium bromide CNTs were marginally responsive toward tested VOCs but were not selective. In contrast, pure CNTs responded well to most of the tested aromatic VOCs. The selective responsiveness was attributed to the nature of the surfactant and the interaction between the surfactant remnants as dopants with the CNT system. The study proposed the mechanism of controlling the selectivity of CNT-based sensors toward the desired biomarkers by optimizing the nature and concentration of surfactants in accordance with their critical micelle concentrations.

Further, the CNT was functionalized with organic molecules to enhance its selectivity and sensitivity toward specific breath biomarkers. Nag et al.^179^ fabricated a polyhedral oligomeric silsesquioxane (POSS) functionalized CNT-based chemoresistive array to detect acetone in human breath selectively (Figure 8B). The outcomes suggested a unique method to optimize the cessation of the nanoheterojunctions of the percolated conducting network in a chemiresistor for enhanced breath biomarker sensing. In another study,^180^ they functionalized hybridized CNT and fullerene using poly(ether ether ketone) for detecting VOCs present in exhaled breath up to a subppm level (as low as 340 ppb). Moreover, the fabricated E-nose, which especially senses methanol, has a high SNR of around 200 among all tested biomarkers.

Besides, Kumar et al.^181^ tailored the physicochemical attributes of CNTs by functionalization with polyelectrolyte systems. They utilized an electrostatic layer-by-layer assembly technique to fabricate 16 bilayers of sodium deoxycholate (DOC)/poly(diallyl dimethylammonium chloride) CNTs, providing optimum chemoresistive attributes to detect and distinguish eight VOCs, including chloroform, water, ethanol, toluene, dichloromethane, acetone, methanol, and tetrahydrofuran. The cationic polyelectrolyte PDDA contributed to augmenting CNT sensing response toward VOCs. Consequently, this method has robust latent progress in fabricating highly sensitive VOC chemiresistors for LC screening.

These experimental investigations are also supported with theoretical modeling and simulations, which can be performed before experimental evaluations to confirm NM’s suitability for specific target analytes and save human resources and environmental contamination. For instance, Aasi et al.^182^ theoretically demonstrated the potential of platinum-group transition metal (Pt, Ru, Rh, or Pd) decorated SWCNTs as prospective nanosensors for the toluene monitoring, which is a vital VOC in the LC patient’s exhaled breath. DFT studies revealed the physisorption of the toluene molecule on SWCNTs by the interaction of the nanotube and the π-orbitals of the toluene’s carbon atoms. However, covering the SWCNT with transition metals improved the adsorption energies considerably, giving the higher sensitivities (−96.98% and −99.98%, respectively) through robust overlapping among p-orbitals of C atoms in the toluene’s benzene ring and d-orbitals of the metal atoms. These findings demonstrated transition metal decorated SWCNTs for toluene detection, supporting breath-based LC diagnosis.

The application of carbon-based NMs in detecting LC biomarkers from exhaled breath has extended beyond CNTs to include graphene and its derivatives in recent years. Majidi and Nadafan^183^ conducted theoretical investigations to explore the potential of graphene derivatives in detecting LC biomarkers. Using DFT calculations, they examined the adsorption behavior of typical LC breath biomarkers, such as benzene, *o*-toluidine, styrene, and aniline, on twin-graphene and γ-graphene sheets (Figure 8C). The results revealed that the energy band gaps of both graphene variants decrease upon adsorbing VOCs, and the weak binding energy between graphene derivatives and the tested VOCs makes them suitable for developing fast, recoverable, and repeatable sensors. Furthermore, twin graphene exhibits enhanced electronic properties compared to γ-graphyne, making it a more promising candidate for experimental evaluation in LC biomarker detection from exhaled breath.

These findings were further supported by experimental evaluations of graphene and its derivatives. Chen et al.^184^ conducted an experimental study to assess the capabilities of a rGO sensor induced by metal ions in detecting four specific biomarkers associated with LC, including acetone, isoprene, ammonia, and H_2_S. Their findings demonstrated that using linear regression analysis on data collected from a study involving 106 participants it was possible to distinguish between the healthy group and LC patients accurately. By incorporating an artificial neural network, the E-nose achieved an impressive sensitivity of 95.8% and specificity of 96.0% for diagnosing LC. The improved sensitivity can be attributed to the added interaction among the VOC and the metal species. These results indicate the significant potential of the proposed carbon-based E-nose for noninvasive disorder/infection detection and personalized healthcare management.

Moreover, the functionalization of rGO improves its selectivity by providing the desired surface functionalities for binding particular analytes. For instance, Nag et al.^185^ developed an E-nose by wrapping rGO with functionalized β-cyclodextrin, resulting in supramolecular assembly for detecting VOCs associated with LC screening. The host–guest interaction resulted in improved electrical conductivity, augmented surface area, complex formation ability, and tunable chemical functionality, and it exhibited excellent LC screening by detection of its breath biomarkers.

### Molecularly Imprinted Polymer-Enabled Nanosensors for Breath-Based Diagnosis of Lung Cancer

Molecularly imprinted polymer (MIP)-based chemiresistive sensors have emerged as promising tools for screening LC through the analysis of human breath samples.^186^ These sensors are designed to replicate the specific recognition properties of antibodies or receptors by imprinting target molecules onto a polymer matrix. When a breath sample containing VOCs associated with LC is introduced into the MIP sensor, the VOCs selectively bind to the imprinted sites within the polymer. This binding process induces a change in the electrical resistance of the sensor. By measuring this variation in resistance, one can correlate it with the presence and concentration of VOCs, thereby providing valuable information for the early detection of LC. For example, Mousazadeh et al.^186^ fabricated a sensor based on Au NP-enhanced MIP for low trace detection of breath hexanal for LC screening (Figure 8D). The sensor was also evaluated in the electrochemical module to detect hexanal in various biological matrices, including serum, urine, saliva, plasma, and cell cultures (Figure 8E). The sensor exhibited low LOD as low as 1.1 ppm, high selectivity, and a linear detection range of 2.5–300 ppm, which portrays its potential in detecting hexanal for LC screening (Figure 8F).

In addition, the sensing performance of MIPs can be improved through the functionalization of other NMs. For instance, Janfaza et al.^187^ developed a chemiresistor by combining MIP nanoparticles with multiwalled CNTs (MWCNTs) for the sensitive monitoring of trace levels of breath hexanal in LC screening. The sensor demonstrated functionality within the 10 to 200 ppm concentration range, with an LOD of 10 ppm and an SNR of approximately 3. Remarkably, the sensor exhibited robust recovery and high repeatability, returning to its initial state without heating, even at high hexanal concentrations. These findings highlight the potential for designing commercial sensors for LC screening that offer reliable performance and recovery.

### Advanced Nanocomposite Enabled Sensors for Breath-Based Diagnosis of Lung Cancer

Nanocomposite (NC)-enabled sensors have emerged as a promising platform for BBD of LC. By integrating NMs, such as MOs or CNTs, with other functional components, these sensors offer enhanced sensitivity and selectivity for VOCs associated with LC. The synergistic effects due to the formation of heterojunctions such as p-n/p-p junctions, advanced morphologies such as core–shell structure, and desired surface optimization as per targeted analytes make NCs a high-performance platform for VOC monitoring to design breathomic-based LC screening technologies.^150,152,188−190^

Chatterjee and colleagues^191^ have developed an E-nose utilizing quantum chemiresistors based on CNTs dispersed in a conducting polymer matrix. This E-nose was designed to detect various VOCs selected as LC biomarkers. The VOCs included a set of polar vapors such as water, ethanol, methanol, acetone, propanol, isopropanol, and 2-butanone, as well as a set of less and nonpolar vapors including chloroform, toluene, benzene, styrene, cyclohexane, *o*-xylene, *n*-propane, *n*-decane, 1,2,4-trimethylbenzene, isoprene, and 1-hexene. The quantum chemiresistors showed great potential as cost-effective E-nose components for diagnosing LC through VOC analysis in breath. They exhibited sensitivity at the parts per million level, with sensitivity tested down to 2.5 ppm. The response time was rapid, typically a couple of seconds, and the devices had a low power consumption. Additionally, the SNR was high, with a value of ≥10. Furthermore, PCA demonstrated excellent recognition capabilities by distinguishing between the different biomarkers and their concentrations, enabling the identification of subjects.

**Figure 9:**
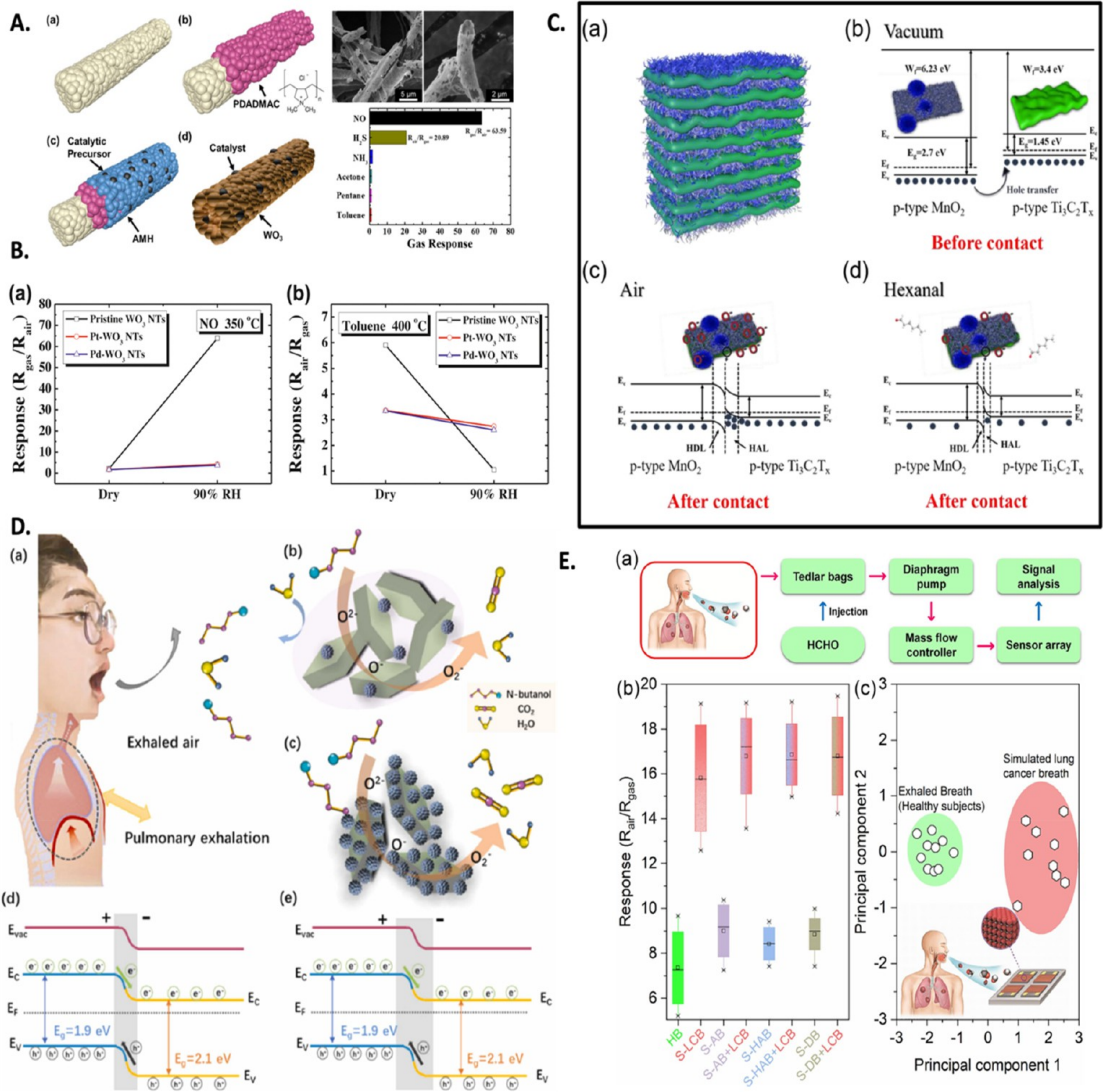
Chemiresistors based on advanced nanocomposites, including (A) the effect of catalyst doping on WO_3_ nanotubes’ morphology and LC breath biomarker-based sensing characteristics and (B) comparison of pristine WO_3_ and Pt/Pd-doped WO_3_ nanotubes for detecting NO and toluene at elevated temperatures for LC diagnosis from human breath. Subfigures A and B are reproduced from ref (192). Copyright [2016] Elsevier. (C) Schematic illustration of the morphology of MnO_2_/Ti_3_C_2_T_*x*_ NC in stack form, formation of heterojunctions between the two precursors, and modulation of the depletion layer form at the heterojunction of precursors in ambient surrounding/hexanal to evaluate LC diagnosis characteristics, reproduced with permission from ref (193). (D) Schematic representation of detection of LC biomarkers from exhaled breath of humans and their interaction with advanced (α-Fe_2_O_3_/ZnFe_2_O_4_) nanoheterojunction NCs leading to modulation in depletion layer formed at the junction of two precursors. Reproduced from ref (195). Copyright [2023] Elsevier. (E) Schematic representation of steps included in evaluating the point-of-care sensing performance of the NC chemiresistor for detecting LC biomarkers in human breath, including storing exhaled breath of patients in Tedlar bags and pumping them into sensing chambers possessing a sensor array, which records the variation of resistance of NCs for different biomarkers. Various sensing signals were distinguished using PCA analysis to differentiate among LC biomarkers and environmental factors. Reproduced from ref (197). Copyright [2022] Elsevier.

In their research, Koo et al.^192^ investigated the detection performance of a sensor based on highly porous WO_3_ nanotubes decorated with catalysts and loaded with an ionic polymer in poly(methyl methacrylate) (PMMA) for identifying LC breath biomarkers (Figure 9A). The pristine WO_3_ nanotubes responded significantly to NO at 350 °C (sensing response = 63.59 at 5 ppm) and demonstrated cross-selectivity toward toluene (sensing response = 1.05 at 5 ppm) (Figure 9B). In contrast, the Pt-WO_3_ and Pd-WO_3_ nanotubes showed a high response to toluene at 400 °C (sensing response = 2.24 and 2.35 for Pt-WO_3_ and Pd-WO_3_ at 5 ppm, respectively) but a negligible sensing response to NO at the same temperature (sensing response = 1.25 and 1.04 for Pt-WO_3_ and Pd-WO_3_ at 5 ppm, respectively) (Figure 9B). These results suggest that modifying the catalyst makes it possible to achieve the desired selectivity for the detection of LC biomarkers. Additionally, hollow structures with optimal porosity enhance the adsorption of biomarker molecules, leading to stronger sensing signals and making them highly suitable for developing LC biomarker detection platforms.

In a recent study, Yao et al.^193^ presented a new approach to LC screening, focusing on monitoring hexanal using an optimized sensor based on manganese dioxide (MnO_2_)/Ti_3_C_2_T_*x*_ NCs (Figure 9C). The sensor exhibited a remarkable sensitivity of approximately 52%, a low LOD of 20 ppm, and rapid recovery times of around 134 s. The key contributor to the detection capabilities of the NC sensor was identified as MnO_2_, which served as the active center due to its high reactivity and anoxia, specifically targeting hexanal molecules. The presence of p–p heterojunctions at the interface of MnO_2_ and Ti_3_C_2_T_*x*_ within the NC structure further enhanced the sensitivity of the sensor. Additionally, the catalytic activity of both precursors played a vital role in providing the NC-based sensor with a unique selectivity. These findings demonstrate the potential of NCs based on emerging 2D NMs in developing chemiresistive modules for BBD of LC. Through their synergistic effects and optimized morphologies, these NCs hold promise for advancing the field of LC detection.

Recent research has focused on evaluating magnetic nanosystems and nanoheterojunctions for LC screening through breath analysis. For instance, Zheng et al.^194^ evaluated the LC screening potential of green synthesized bismuth ferrite (BiFeO_3_) NP-based sensors through low-trace breath isopropanol detection. The gas sensor utilizing BiFeO_3_ showcased exceptional sensitivity when operated at an optimized temperature of 275 °C. It displayed a robust linear relationship between the sensor’s response and the concentration of the gas present. Remarkably, even in an environment with 100% RH, the sensor achieved a response value of 3.9 when exposed to 1 ppm of isopropanol. The magnetic attributes surge the detection efficacy of the sensor and selective isopropyl alcohol detection at low concentrations. Recently, Yan et al.^195^ developed an E-nose utilizing hematite/franklinite (α-Fe_2_O_3_/ZnFe_2_O_4_) nanoheterojunctions with strong magnetism to detect trace levels of *N*-butanol in exhaled breath, which serves as a potential LC biomarker (Figure 9D). The magnetic nature of the fabricated nanostructures enables efficient oxygen capture for redox reactions, and the presence of n–n nanoheterojunctions significantly enhances the sensing response, thus improving sensitivity even at concentrations as low as 0.1 ppm and operating temperatures of 160 °C. The nanostructures exhibited negligible sensitivity reduction in the presence of high humidity, with a minimal decrease in the sensing response even at 91% relative humidity. This humidity insensitivity and strong magnetism make these nanostructures suitable for developing stable and practical sensors for LC screening based on breath analysis.

The detection of LC biomarkers in human breath is also extended to monitoring diversified VOCs using NC-based POC sensors, and in a recent study, Shanmugasundaram et al.^196^ developed an E-nose using an NC superstructure composed of rGO and SnO_2_ to detect formaldehyde (HCHO) with high sensitivity specifically. The sensor exhibited a low LOD of 10 ppb for HCHO at an operating temperature of 125 °C. Since the concentration of HCHO in the breath of LC patients is typically higher (83 ppb) compared to that of healthy individuals (48 ppb), the fabricated E-nose successfully distinguished between breath samples of healthy subjects and those with LC using PCA investigations. The exceptional performance of NC in detecting LC biomarkers can be attributed to its unique superstructure, optimized stoichiometry, and synergistic effects. In another investigation,^197^ the research team employed an rGO-incorporated SnO_2_ nanosphere-based sensor to monitor decane and heptane in human breath for LC diagnosis (Figure 9E). The fabricated chemiresistor exhibited exceptional sensitivity to heptane and decane, outperforming other interfering analytes present in breath with a low LOD of 1 ppm and rapid response/recovery at 125 °C. The proposed sensor offers a simple and effective screening method for LC patients by detecting the presence of decane and heptane in their exhaled breath. Consequently, the various choices and combinations of materials form NCs with optimized properties for the selective detection of breath biomarkers for LC screening.

## Challenges, Alternative Solutions, and Future Directions

The challenges and future directions in the field of sensor-based breath analysis for LC diagnosis are discussed in this section. It addresses issues such as standardization of sampling protocols, integration of sensor technologies into existing clinical workflows, and the need for large-scale multicenter studies. Additionally, this study explores the potential for combining breath analysis with other diagnostic modalities and integrating AI and ML algorithms for enhanced diagnostic accuracy.

### Manufacturing, Processing, and Operational Concerns with Alternative Solutions

Developing sensors based on NMs for detecting LC through breath analysis presents several challenges and risks that must be addressed. One of the primary challenges is designing sensors with high sensitivity and selectivity to detect specific biomarkers associated with LC.^64,198−205^ Differentiating cancer-related biomarkers/VOCs from background compounds in breath is complex. NMs, with their unique properties such as high surface-to-volume ratio and enhanced reactivity, hold the promise of achieving better sensitivity and selectivity. However, the challenge lies in optimizing NMs to selectively bind and detect target biomarkers while minimizing interference from other compounds. Various techniques, such as functionalization with specific biochemical or functionalities to bind targeted biomarkers, must be explored to enhance the sensitivity and selectivity of breath biomarkers.^206−209^ Moreover, integrating advanced data/pattern analytics, AI, and bioinformatics techniques can address these challenges for distinguishing various sensing signals emerging from diversified breath biomarkers of LC.^82,210,211^

Ensuring the stability and long-term functionality of NM-based sensors is also crucial. NMS may degrade or undergo structural changes over time, decreasing the sensor performance. Factors such as environmental conditions, exposure to moisture, and interactions with biomolecules can affect their stability.^27,212−215^ Strategies should be developed to enhance the stability and longevity of sensors to ensure the accurate and reliable detection of lung cancer biomarkers. For instance, coating, inert surface doping/functionalization, and innovative morphologies (such as core–shell) of sensor surfaces must be explored to attain the long-term stability of these sensors.^216−220^ NM-based breath sensors for LC diagnosis require standardization and reproducibility to facilitate widespread adoption and commercialization. Achieving consistency in sensor fabrication and performance across different manufacturing processes and research groups is a significant challenge. Developing standardized protocols, implementing quality control measures, and establishing validation procedures are necessary to ensure the reliability and comparability of sensor data generated from various sources. By incorporating all of the literature and designing standard protocols with advanced data analytics, standardization can be achieved in fabricating and utilizing these sensors.

### Biocompatibility, Toxicity, and Safety Concerns with Alternative Solutions

Integrating NMs into breath-based sensors necessitates thoroughly evaluating their biocompatibility, toxicity, and potential safety risks. Some NMs may exhibit toxicity or elicit immune responses in the human body because of leaching out or inhaling NMs into the human body. Toxicity is a significant concern when using NMs in medical applications.^5,221−224^ Certain NMs, such as MB-NPs or CNTs, may possess inherent toxicity and can cause cellular damage or inflammation when exposed to biological systems.^225,226^ Therefore, it is crucial to carefully select NMs with low toxicity profiles for breath sensors. Researchers should conduct toxicity studies to assess the safety of these NMs and establish concentration limits to mitigate potential risks.

Biocompatibility is another crucial factor to consider when utilizing NMs for medical diagnostics. NMs used in breath sensors must be biocompatible, meaning they should not cause adverse effects or trigger immune responses in the human body.^134,227−229^ Comprehensive testing should be conducted to evaluate the interaction of NMs with cells, tissues, and biological fluids. Applying surface modifications or coatings can enhance biocompatibility and minimize potential adverse effects.^134,228,229^ Besides, safety risks associated with NM-based breath sensors include device malfunction, sample contamination, and exposure to hazardous chemicals during fabrication or operation. Implementing quality control measures, following standardized protocols, and conducting rigorous safety assessments are essential to mitigate these risks. This ensures consistent performance, minimizes variability and maintains safety in clinical settings. Moreover, the large-scale utilization of these sensors raises the issue of solid-waste generation after usage and chemical-based environmental contamination during fabrication.^134,228^ The improper disposal of the byproducts during fabrication and of sensors after utilization can also lead to the leaching of NMs into the food chain and ecosystem, affecting the lives of numerous flora and fauna and environmental/climatic integrity.

Several steps can be taken to address these concerns and enhance the safety of NM-based breath sensors for LC diagnosis. First, the selection of NMs should be based on thoroughly evaluating their toxicity profiles, prioritizing those with low toxicity and proven biocompatibility. Surface modifications or coatings can then be applied to improve the biocompatibility and reduce potential toxicity. Conducting comprehensive toxicity studies, both in vitro and in vivo, is crucial to assess the potential adverse effects of NMs.^5,222,230^ To ensure their safety, these studies should evaluate their impact on cells, tissues, and animal models. Furthermore, strict quality control measures should be implemented throughout the fabrication, assembly, and testing processes to ensure a consistent and safe performance of the breath sensors. Developing standardized protocols for operating and maintaining NM-based breath sensors is essential to minimizing variability and maintaining safety in clinical use. Compliance with relevant regulatory guidelines and seeking approvals are also essential to meet safety and ethical standards. Alternatively, adopting green strategies, such as using green NMs and surfaces, can effectively address the issues of toxicity, biocompatibility, and safety risks associated with NM-based breath sensors for LC diagnosis. Green strategies focus on minimizing environmental impact and promoting sustainable practices throughout the product’s lifecycle.^134,228,229,231,232^ One approach to address these concerns is by using green NMs. These NMs are designed to be environmentally friendly and have low toxicity, reducing the risk of adverse effects on human health and the environment.^10,232,233^ The inherent toxicity concerns can be mitigated by incorporating green NMs into breath sensors.

Another aspect is the implementation of green surfaces. Surface modifications play a crucial role in enhancing the biocompatibility and reducing toxicity. Green surface engineering involves using environmentally friendly processes and materials to modify the surfaces of the NMs. This approach improves the biocompatibility of breath sensors and minimizes potential adverse interactions with biological systems.^134^ Moreover, sustainable production practices are an essential component of green strategies. It involves adopting eco-friendly manufacturing processes, minimizing hazardous chemicals, reducing waste generation, repurposing/reusing/recycling generated waste, and adopting waste-to-wealth modules.^134,234−236^ By the implementation of sustainable production methods, the risk of exposure to harmful chemicals during fabrication or operation can be significantly reduced.

Furthermore, conducting a life cycle assessment (LCA) is another valuable step. An LCA evaluates the environmental impact of NM-based breath sensors throughout their entire life cycle, from raw material extraction to disposal. This assessment considers resource consumption, energy use, and waste generation. By identifying environmental hotspots and implementing improvements, breath sensors’ overall safety and sustainability can be enhanced. Besides, the proper disposal and management of NMs after use should also be considered to prevent environmental contamination. Similarly, compliance with regulatory frameworks and guidelines is vital in green strategies. These regulations promote the safe and responsible use of NMs, ensuring that breath sensors meet safety standards and are designed to minimize risks to both patients and the environment. Adhering to regulatory requirements ensures that the sensors are developed and used safely and sustainably.

Consequently, adopting green strategies, including using green NMs, green surfaces, sustainable production practices, life cycle assessments, and regulatory compliance, can effectively address the concerns related to toxicity, biocompatibility, and safety risks associated with NM-based breath sensors. These strategies promote the development of safer and more sustainable technologies for LC diagnosis, contributing to a healthier environment and protecting human health. In summary, the safety concerns associated with NM-based breath sensors, including toxicity, biocompatibility, and general safety risks, can be effectively addressed by following these steps. It paves the way for their responsible and safe use in the BBD of LC, benefiting patients and healthcare providers alike.

### Clinical Validation, Regulatory Approval, Ethical, and Privacy Considerations

The transition of NM-based breath sensors from the lab to clinical practice requires rigorous clinical validation and regulatory approval. Conducting large-scale clinical trials to assess the sensors’ sensitivity, specificity, and accuracy is complex and resource-intensive.^6,141,237^ Additionally, obtaining regulatory approvals from relevant authorities, such as the Food and Drug Administration (FDA), adds further challenges and time constraints to the development process. The collection and analysis of personal health data through NM-based breath sensors raise ethical and privacy concerns. Protecting patient privacy, ensuring informed consent, and addressing data ownership and security are crucial aspects that need careful attention. Robust data protection mechanisms and adherence to established ethical guidelines are necessary to maintain patient trust and ensure the responsible implementation of this technology. Thus, NM-based sensors for breath-based lung cancer diagnosis offer great potential. However, overcoming challenges related to sensitivity, stability, standardization, safety, clinical validation, and ethical considerations is necessary for a successful implementation. By addressing these hurdles, NM-based sensors can significantly improve lung cancer diagnostics, leading to earlier interventions and improved patient outcomes.

### Future Directions and Modern-Age Integrations

The potential for transforming NM-based sensors into a commercially viable and practical technology for BBD of LC lies in the integration of modern-age technologies and in pursuing specific future directions. These include continued research and development, adoption of AI, ML, IOT, wearable, and POC modules, data integration, and health informatics.^17,64,95,111,113,134,238,239^ Additionally, establishing a regulatory framework and fostering partnerships across different sectors are crucial to successful commercialization. Investing in ongoing research and development is essential to enhance the sensitivity, selectivity, stability, and reproducibility of NM-based sensors used in lung cancer detection. Exploring innovative NMs, such as 2D materials, hybrids, and composites, can significantly improve the performance of these sensors.^95,240,241^ Furthermore, investigating scalable manufacturing techniques, including additive manufacturing, can enable cost-effective and large-scale production. MI/AI algorithms should be employed to analyze the complex data generated by NM-based sensors and develop accurate predictive models for diagnosis and monitoring. Integrating these algorithms with the sensors allows for real-time data analysis and interpretation. Moreover, incorporating IOT connectivity into NM-based sensors facilitates seamless data transfer and remote monitoring. Cloud-based platforms can be utilized for efficient data storage, analysis, and collaboration among healthcare providers and researchers.

Designing wearable and portable breath sensor devices that seamlessly integrate into everyday life enables continuous monitoring and early detection of LC.^54,242−245^ Developing POC devices for rapid on-site analysis of breath samples can provide immediate feedback and reduce diagnosis turnaround time.^77,109,213^ Integrating data from NM-based sensors with electronic health records and other clinical data can create comprehensive patient profiles and facilitate personalized medicine approaches. Health informatics techniques can extract valuable insights from large data sets, aiding in early detection, prognosis, and treatment optimization. Establishing clear regulatory guidelines and standards for NM-based breath sensors ensures safety, efficacy, and interoperability. Collaboration with regulatory agencies streamlines the approval process and addresses the commercialization challenges. Partnerships with healthcare institutions, industry stakeholders, and investors are crucial for translating research into commercially viable products. Moreover, integration with advanced sixth-generation technologies, including 6G networks and holography, can revolutionize the field of LC diagnostics with these nanobiosensors composed of NOC modules. By focusing on these future directions and integrating modern-age technologies, NM-based sensors for BBD of LC can be advanced further, making them commercially available, practical, and beneficial for patients, healthcare providers, and the healthcare industry.

## Outlook

**Figure 10:**
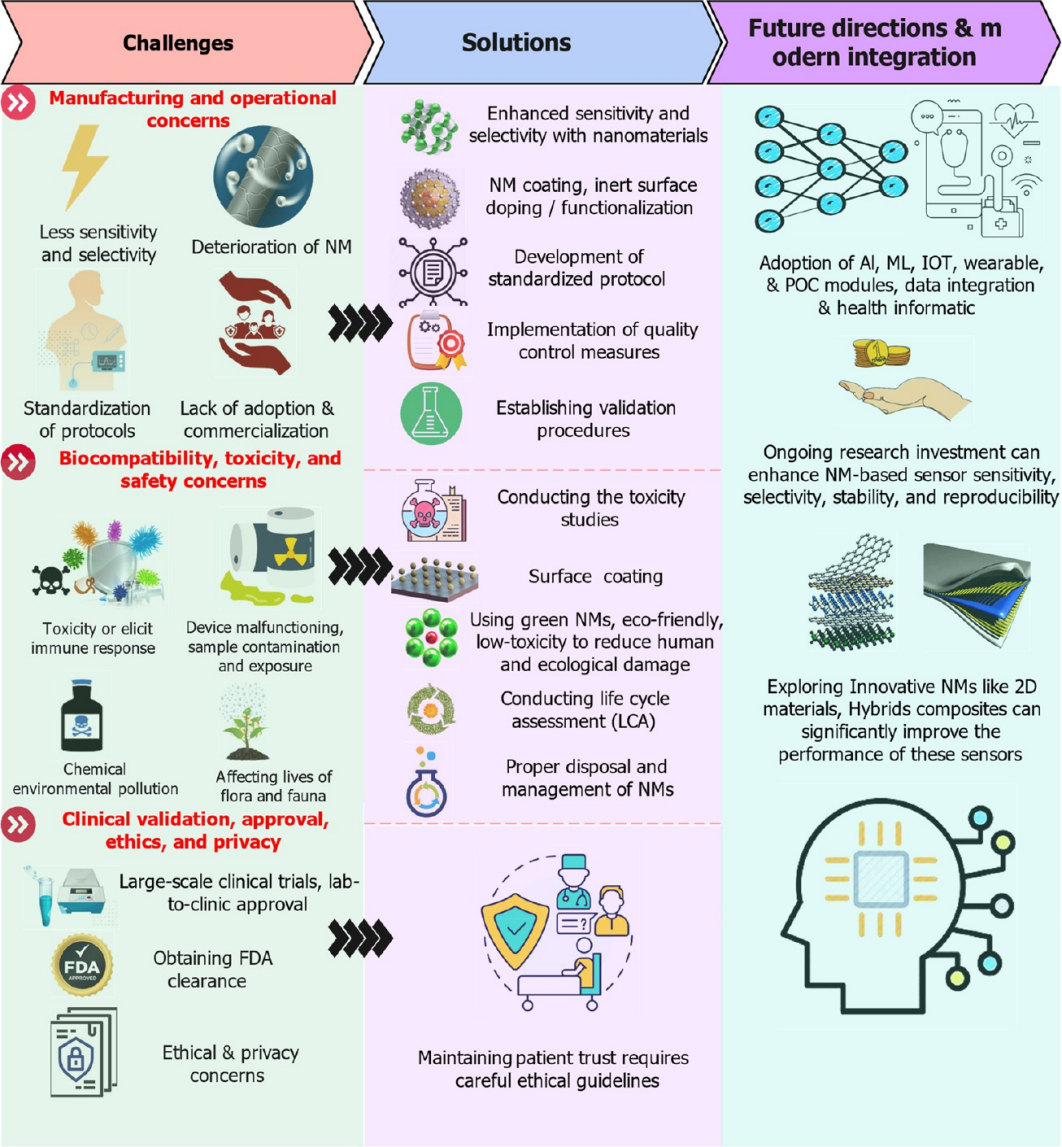
Challenges and alternate solutions related to the adoption of breathomic biosensors based on nanomaterials in the chemiresistive module with the need for clinical validation, regulatory protocols, and ethical considerations. Prospects of these sensors by integrating modern-age technologies have been summarized.

This review summarizes the current state of sensor-based breath analysis for lung cancer diagnosis and its potential impact on clinical practice. It highlights the advantages of sensors in facilitating accurate and real-time detection of lung cancer, offering a promising avenue for noninvasive diagnosis and personalized management using Nose-on-chip module nanobiosensors. The review underscores the importance of further research, standardization, and validation to enable the successful translation of sensor-based breath analysis into routine clinical use. In conclusion, nanomaterial-based sensors for diagnosing lung cancer show great promise for revolutionizing early detection and disease monitoring. The review of current research and advancements in Nose-on-chip nanobiosensors for breath-based lung cancer diagnosis reveals significant progress. It identifies challenges that must be overcome for a successful implementation. Nanomaterials possess unique properties, such as high surface-to-volume ratio, enhanced reactivity, and tunable characteristics that make them well-suited for sensing applications. These sensors have the potential to provide high sensitivity and selectivity in detecting lung cancer biomarkers present in breath samples. However, addressing challenges related to sensitivity, stability, standardization, biocompatibility, clinical validation, and ethical considerations is crucial (Figure 10).

To tackle these challenges, future research directions should focus on exploring alternative nanomaterials, developing surface functionalization techniques, adopting green strategies, and implementing sensor arrays to enhance sensitivity and selectivity. Stability can be improved through protective coatings and inherently stable nanomaterials. Standardized protocols and collaboration among research groups can promote reproducibility and facilitate the validation of sensor performance (Figure 10). It is imperative to conduct comprehensive biocompatibility studies and toxicity assessments to ensure the safety of the nanomaterials used in these sensors. Large-scale clinical trials are essential to validate the effectiveness of these sensors in diagnosing lung cancer. Collaboration with regulatory agencies is necessary to navigate the approval process and establish regulatory guidelines. Furthermore, incorporating modern-age technologies like ML, IOT connectivity, holography, 5G/6G communication, and health informatics can enhance sensor capabilities, data analysis, and integration into existing diagnostic processes. By addressing these aspects, nanomaterial-based sensors for lung cancer diagnosis can become commercially viable and widely adopted. This technology can potentially transform lung cancer diagnostics, enabling early detection, personalized treatment approaches, and improved patient outcomes. Continued advancements in the development of these Nose-on-chip nanobiosensors bring us closer to a future where lung cancer can be detected at its earliest stages, leading to more effective interventions and ultimately to save lives.

## Vocabulary

Breathomics: the study and analysis of volatile organic compounds (VOCs) and other metabolites present in exhaled breath to assess health and disease states. This emerging field combines principles from metabolomics, analytical chemistry, and medical science to noninvasively monitor physiological conditions and diagnose diseases
VOCs: organic chemicals that easily vaporize at room temperature. They are emitted from various sources, including humans, plants, animals, and even industrial processes, and can serve as biomarkers for health and environmental assessments
Nose-on-chip: a sensor designed to mimic the human olfactory system, detecting and identifying complex mixtures of VOCs
LC screening: lung cancer (LC) screening refers to the process of detecting lung cancer at an early, more treatable stage in individuals who are at high risk for the disease, particularly long-term smokers and older adults
Chemoresitive sensors: a type of sensor used to detect chemical substances based on changes in electrical resistance. These sensors typically use materials whose electrical resistance changes when interacting with specific target molecules, such as volatile organic compounds (VOCs), gases, or other chemicals
Biomarkers: measurable indicators of biological processes, conditions, or diseases. They are often used in clinical and research settings to assess health, diagnose diseases, monitor disease progression, and evaluate responses to treatments.
